# Application of a Physiologically Based Pharmacokinetic Model to Predict Cefazolin and Cefuroxime Disposition in Obese Pregnant Women Undergoing Caesarean Section

**DOI:** 10.3390/pharmaceutics14061162

**Published:** 2022-05-30

**Authors:** Hanadi H. Alrammaal, Khaled Abduljalil, Victoria Hodgetts Morton, R. Katie Morris, John F. Marriott, Hsu P. Chong, Hannah K. Batchelor

**Affiliations:** 1 School of Pharmacy, Institute of Clinical Sciences, University of Birmingham, Birmingham B15 2TT, UK; j.f.marriott@bham.ac.uk; 2 Clinical Pharmacy Department, Collage of Pharmacy, Umm Al-Qura University, Mecca 24382, Saudi Arabia; 3 Simcyp Division, Certara UK Limited, Sheffield S1 2BJ, UK; khaled.abduljalil@certara.com; 4 Department of Fetal and Maternal Medicine, Birmingham Women’s and Children’s NHS Foundation Trust, Birmingham B15 2TG, UK; v.a.h.morton@bham.ac.uk (V.H.M.); r.k.morris@bham.ac.uk (R.K.M.); 5 Institute for Metabolic and Systems Research, University of Birmingham, Birmingham B15 2TT, UK; 6 Rosie Maternity Hospital, Robinson Way, Cambridge CB2 0SW, UK; hsu.chong@nhs.net; 7 Strathclyde Institute of Pharmacy and Biomedical Sciences, University of Strathclyde, Glasgow G4 0RE, UK; hannah.batchelor@strath.ac.uk

**Keywords:** cefuroxime, cefazolin, physiologically-based pharmacokinetic model, caesarean section, obese pregnant, adipose exposure

## Abstract

Intravenous (IV) cefuroxime and cefazolin are used prophylactically in caesarean sections (CS). Currently, there are concerns regarding sub-optimal dosing in obese pregnant women compared to lean pregnant women prior to CS. The current study used a physiologically based pharmacokinetic (PBPK) approach to predict cefazolin and cefuroxime pharmacokinetics in obese pregnant women at the time of CS as well as the duration that these drug concentrations remain above a target concentration (2, 4 or 8 µg/mL or µg/g) in plasma or adipose tissue. Cefazolin and cefuroxime PBPK models were first built using clinical data in lean and in obese non–pregnant populations. Models were then used to predict cefazolin and cefuroxime pharmacokinetics data in lean and obese pregnant populations. Both cefazolin and cefuroxime models sufficiently described their total and free levels in the plasma and in the adipose interstitial fluid (ISF) in non–pregnant and pregnant populations. The obese pregnant cefazolin model predicted adipose exposure adequately at different reference time points and indicated that an IV dose of 2000 mg can maintain unbound plasma and adipose ISF concentration above 8 µg/mL for 3.5 h post dose. Predictions indicated that an IV 1500 mg cefuroxime dose can achieve unbound plasma and unbound ISF cefuroxime concentration of ≥8 µg/mL up to 2 h post dose in obese pregnant women. Re-dosing should be considered if CS was not completed within 2 h post cefuroxime administration for both lean or obese pregnant if cefuroxime concentrations of ≥8 µg/mL is required. A clinical study to measure cefuroxime adipose concentration in pregnant and obese pregnant women is warranted.

## 1. Introduction

Cefuroxime and cefazolin are widely used as intravenous prophylactic antibiotics in surgical settings [1,2]. The plasma unbound fraction (f_u_) of cefazolin and cefuroxime are different in healthy adults; 0.225 and 0.67, respectively [3,4,5]. While the reported lipophilicity of cefuroxime is lower than cefazolin (Log *p* values of −0.90 vs. −0.58, respectively) [6,7]. Cefuroxime’s apparent volume of distribution (V_d_) is approximately 1.4–fold higher than that of cefazolin in healthy adults [8,9]. Both drugs are cleared entirely by the kidneys [10,11,12,13]. Transporter medicated clearance is believed to be responsible for 50–80% of cefazolin total clearance [14]. For cefuroxime, approximately 45–50% is reported to be cleared by tubular secretion [3].

Physiological and biochemical alterations, observed in pregnancy, can greatly impact drug kinetics [15,16]. The increased incidence of obesity within the pregnant population further complicates predictions of drug kinetics as obesity can also affect drug kinetics [17]. The extent and direction of clinical impacts of both obesity and gestation on pharmacokinetics and pharmacodynamics (PK/PD) has not been clearly established for the majority of drugs. While cefazolin PK/PD in obese pregnant women, body mass index (BMI) ≥ 30 kg/m^2^, has been investigated due to concerns around sub-optimal dosing in this population compared with those of normal body weight (BMI 18.50–24.99 kg/m^2^) at time of caesarean section (CS); to date, there is no information regarding disposition of cefuroxime in an obese pregnant population at time of CS [2,18,19,20,21,22,23,24]. Prophylactic cefuroxime doses used in CS setting are 750 mg or 1500 mg given pre skin incision [1,25,26]. Although the minimum inhibitory concentrations required to inhibit the growth of 90% of organisms (MIC_90_) of cefuroxime and cefazolin for most of the common causative bacteria of post CS infections is 1–2 µg/mL, various bacterial strains may require higher concentrations, i.e., MIC_90_ 2, 4 and 8 µg/mL [19,27].

The physiologically-based pharmacokinetics (PBPK) approach has the potential to predict drug exposure in pregnant women based on the drug’s physiochemical properties and knowledge of physiological changes during pregnancy [28]. A hypothesis here is, “Will the non–obese pregnancy PBPK approach be able to predict cefazolin and cefuroxime pharmacokinetics in obese pregnant women if known physiological changes in obese subjects are accounted for in the baseline model for non–pregnant subjects?”. This can help to derive clinically relevant conclusions with respect to dosage optimisation for obese pregnant women prior to CS. Therefore, the aims of this study were: (1) to develop PBPK models for cefuroxime and cefazolin in non–pregnant, non–obese subjects as well as in obese non–pregnant subjects using available clinical data of these drugs in the serum and adipose tissue; (2) to use the developed model to predict the exposure in non–obese pregnant and in obese pregnant populations; and (3) to compare simulated plasma and adipose tissue concentrations of different doses to a range of MIC_90_ (i.e., 2, 4 and 8 µg/mL) [19,27], and assess the timing of administration relative to skin incision to ensure that concentrations are >MIC_90_ at the time of incision and during CS. This report follows the checklist suggested for pharmacometric studies [29].

## 2. Materials and Methods

### 2.1. Model Building

Both cefazolin and cefuroxime disposition were investigated in:

(a) Non–obese (lean), non–pregnant (BMI 18.50–24.99 kg/m^2^),
(b) Obese non–pregnant (BMI ≥ 30 kg/m^2^),
(c) Pregnant non–obese (lean pregnant) (BMI 18.50–24.99 kg/m^2^) and
(d) Obese pregnant (BMI ≥ 30 kg/m^2^) populations.

As in vivo cefuroxime disposition data was unavailable for obese pregnant (BMI ≥ 30 kg/m^2^) populations, cefazolin was selected to build a new obese pregnant model; then, cefuroxime concentration in obese pregnant women was simulated using the developed obese pregnant population model.

The Simcyp Simulator (Simcyp Version 20 Release 1) was used. The compound files were developed using a full PBPK (multi-compartment) distribution model and the volume of distribution at steady state (V_SS_) was predicted within the Simcyp simulator using the Rodgers and Rowland method for calculating tissue-to-plasma partition coefficients [30]. To predict the free cefazolin and cefuroxime concentrations in the adipose interstitial fluid (ISF), the generic permeability limited model within the simulator was used for describing the distribution of the drug into the adipose tissue (Equations (1) and (2)),

(1) $$V_{\mathrm{ISF}}\frac{{\mathrm{dC}}_{\mathrm{ISF}}}{\mathrm{dt}}=Q_{\mathrm{Adip}}\left(C_{\mathrm{plasma}}-C_{\mathrm{ISF}}\right)+{\mathrm{CL}}_{\mathrm{PD}}\;\left({\mathrm{Cu}}_{\mathrm{IW}}^{\mathrm{Adip}}-{\mathrm{Cu}}_{\mathrm{ISF}}\;\right)$$

(2) $$V_{\mathrm{IW}}\frac{{\mathrm{dC}}_{\mathrm{IW}}}{\mathrm{dt}}={\mathrm{CL}}_{\mathrm{PD}}\;\left(C_{\mathrm{ISF}}{\mathrm{fu}}_{\mathrm{ISF}}-{\;C}_{\mathrm{IW}}{\mathrm{fu}}_{\mathrm{IW}}\right)$$

where $V_{\mathrm{ISF}}$, $C_{\mathrm{ISF}}$, $V_{\mathrm{IW}}$, and $C_{\mathrm{IW}}$ are the volumes and concentrations of the drug in the ISF and in the intracellular fluid, respectively; $Q_{\mathrm{Adip}}$, is the blood flow to adipose tissue; $C_{\mathrm{Plasma}}$, is the plasma drug concentrations; ${\mathrm{fu}}_{\mathrm{ISF}}$ and ${\mathrm{fu}}_{\mathrm{IW}}$, are the extracellular and intracellular fraction unbound, respectively; and ${\mathrm{Cu}}_{\mathrm{ISF}}$, ${\mathrm{Cu}}_{\mathrm{IW}}$ are unbound ISF and intracellular concentrations, respectively.

The volumes of ISF ($V_{\mathrm{ISF}})\;$and volumes of intracellular fluid ($V_{\mathrm{IW}})$ are 14.1% and 3.9% of the tissue volume, respectively, which is population dependent (i.e., variable prediction of the tissue volume segments which depends on population characteristics) [31]. The extracellular fraction unbound (${\mathrm{fu}}_{\mathrm{ISF}})\;$was assumed to be equal to plasma fu, while the intracellular fraction unbound (${\mathrm{fu}}_{\mathrm{IW}}$) was predicted in the simulator according to published equation [31]. Equations that describe the change of adipose volume and perfusion during pregnancy together with baseline blood flow for the lean and obese baseline populations are given in the supplementary materials. Other baselines values for tissue volumes, compositions, and flows in the model have been published [31].

The glomerular filtration rate (GFR), volume of fluid filtered per unit time from glomerular, is predicted using the modification of diet in renal disease (MDRD) equation [32], which was found to sufficiently predict GFR in obese subjects [33]. The mechanistic kidney permeability model (Mech KiM) was used to account for transporter kinetics (Table 1). Details of the Mech KiM model have been described elsewhere [34,35]. The pregnancy model was coupled with the Mech KiM model as illustrated in Supplementary materials, Figure S1. For predicting the exposure in the obese non–pregnant population, the obese population model within the simulator was used. For predicting the exposure during pregnancy, the pregnancy population model within the simulator was selected. Physiological changes during pregnancy relevant to this model and the way they have been incorporation in the simulator during pregnancy have been published [36,37]. The maternal PBPK model was used without incorporation of the multi-compartmental feto-placental model; this was considered sufficient to serve the aim of this study. Key equations used to predict cefazolin and cefuroxime disposition in lean pregnant, obese, and morbidly obese pregnant women are provided in the Supplementary materials (Equations (S2)–(S13)).

For obese pregnant women, the default non–pregnant physiology of obese or morbidly obese subjects (in terms of tissue blood flows) was used in the simulator as a pre-pregnancy baseline; for which the physiological changes during pregnancy were applied (Supplementary materials Table S1). Any change to the tissue blood flow during pregnancy were kept as pre-defined in the simulator for the pregnant subjects. The pre-pregnancy weight was changed in the population demographic to allow prediction of pregnancy weight (at 39.5 gestational weeks) of either 97.8 Kg (obese pregnant) or 128 kg (morbidly obese pregnant) to reflect weight in obese pregnant subjects.

#### 2.1.1. Physiologically Based Pharmacokinetic Simulation Design

Covariates (age, sex, and gestational age) were set to the mean as reported in the clinical studies. If these covariates were not reported, the default populations’ predefined characteristics values within the simulator were selected (Table 2 and Table 3). If the predicted mean weight was different from the reported mean weight in clinical studies by more than ± 5 kg, the weight within the simulator was adjusted to the mean reported weight ± 5 kg. This was done by adjusting the pre-pregnancy bodyweight code within the Lua script in the population demographic. Simulations were executed after matching the doses to those used in the clinical studies. All virtual studies were set to 20 trials per run.

#### 2.1.2. Evaluation and Refinement of Cefazolin PBPK Model in Lean Non–Pregnant, Obese Non–Pregnant, Lean Pregnant, and Obese Pregnant Subjects

The physiochemical and pharmacokinetic inputs for the PBPK model of cefazolin are shown in Table 1 [53]. Cefazolin is excreted (approximately 100%) as an unchanged drug in the urine [54]. In addition to the glomerular filtration of cefazolin, different transporters are involved in its tubular secretion [10,11,12]. Recently, the activity of renal OAT3 has been shown to increase by approximately 2.2 during the 1st trimester, 1.7 during the 2nd trimester and 1.3–fold during the 3rd trimester [55]. The permeability limited kidney model was used to represent the transporters involved in cefazolin clearance. The gestational age-dependent activity of OAT3 was updated in maternal model according to a recent publication as shown in Equation (3) [53],

(3) $$\begin{array}{c} \mathrm{Renal}\;\mathrm{OAT}3\;\mathrm{pregnancy}\;\left(\mathrm{fold}\;\mathrm{change}\right)\\ \;\;\;\;\;\;\;=1\times \left(1+0.195\times \mathrm{GA}\;-0.0093\times {\mathrm{GA}}^{2}+0.0001154\times {\;\mathrm{GA}}^{3}\right) \end{array}$$

where GA is Gestational age in weeks.

**Figure 1:**
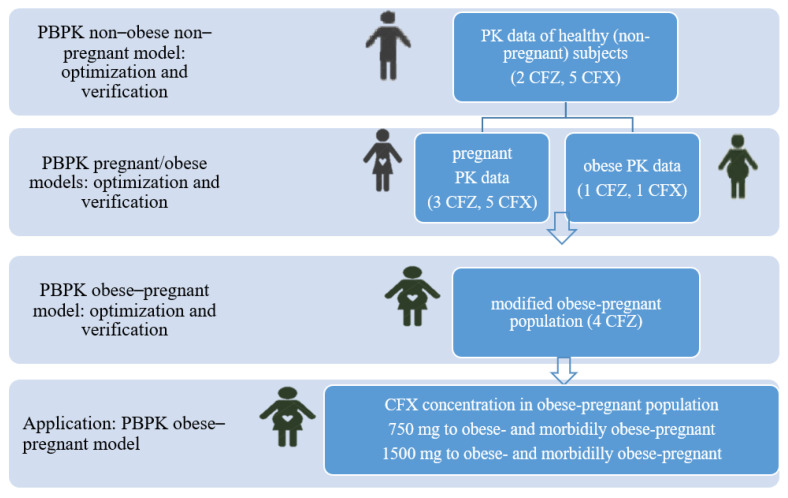
Summary of study optimization, evaluation and application; number in practice represent number of clinical pharmacokinetics data sets used for validation of each drug physiologically-based pharmacokinetics model in an exact population CFX cefuroxime, CFZ cefazolin, PBPK Physiologically-based pharmacokinetics, PK pharmacokinetics.

The PBPK cefazolin model was evaluated using (a) two independent pharmacokinetics studies on lean non–pregnant subjects [8,44], (b) three independent pharmacokinetics studies conducted in pregnant populations [2,8,45,46], (c) one study in an obese population [47] and (d) four independent pharmacokinetics datasets from obese and morbidly obese pregnant populations [21,22,23] (Figure 1).

#### 2.1.3. Evaluation and Refinement of Cefuroxime PBPK Model in Lean Non–Pregnant, Obese Non–Pregnant, and Lean Pregnant Subjects

An existing compound file for cefuroxime has been developed previously by Hsu et al. using the Simcyp simulator version 12.1 in lean non–pregnant subjects [13]. The model was reproducible in version 20. The physiochemical properties and pharmacokinetic parameters of the PBPK cefuroxime model are summarised in Table 1. The predicted Log *p* used in Hsu et al. was −0.9, this was replaced by an experimental value of −1.5 measured in-house using the shake-flask method (Supplementary materials, method Section S1). Despite the fact that the model was not sensitive to the Log *p* change, the measured value was kept in the model for documentation purposes. A global tissues–plasma partition coefficient (Kp) scalar of 1 was used. The Mech KiM model was used to describe the uptake and efflux of active secretion of cefuroxime in the kidney as described by Hsu et al. [13].

The PBPK cefuroxime model was assessed using (a) five pharmacokinetics datasets from lean non–pregnant subjects [9,48,49,50], (b) five observed pharmacokinetics datasets from pregnant populations [1,9,51] and (c) a single set of observed pharmacokinetics data in obese patients who underwent abdominal surgery [52] (Figure 1).

### 2.2. Acceptance Criteria

The PBPK models were considered successful if the simulated plasma area under the curve (AUC) and/or plasma maximum concentration (C_max_) (or first reported time concentration point) were within 2–fold of the observed AUC and/or C_max_. Additionally, visual checks of the observed and simulated concentration–time profiles were performed and considered for accepting the model prediction. Study quality and sample size of studies selected for optimisation of the PBPK models were considered; and any limitation in the study methodology that may affect results of observed cefuroxime and/or cefazolin concentration was countered [56,57]. For all model executions, the reported sample size in each clinical study was replicated in 20 trials of virtual populations.

### 2.3. Application of Obese Pregnant Model to Predict Cefuroxime Disposition

Four clinical scenarios were explored, where 750 mg (study 1) and 1500 mg (study 2) doses of cefuroxime were tested in the obese pregnant population; further, 750 mg (study 3) and 1500 mg (study 4) doses of cefuroxime were tested in the morbidly obese pregnant population. In each scenario, the plasma concentrations were presented for cefuroxime administered at 15, 30, or 60 min before the start of skin incision in CS.

Sources and approaches to analyse constants (or assumptions), covariates and scripted algorithms of selected PBPK models in different populations of interest have been discussed in the literature [30,32,33,34,35,36,37,43]. The scope of the current study is the utilisation of these PBPK models to predict cefazolin and cefuroxime in different populations including the obese pregnant population without the necessity to develop algorithmic equations exclusively for this population; rather using scripted physiological data and pregnancy gestational changes algorithm of the obese population within the simulator.

Due to the unavailability of adipose tissue concentrations in healthy volunteers, the selected models were first “piloted” using observed data of plasma, free plasma, and unbound adipose tissue concentrations of cefazolin and cefuroxime in subjects undergoing surgical procedures.

## 3. Results

### 3.1. Cefazolin PBPK Model

**Figure 2:**
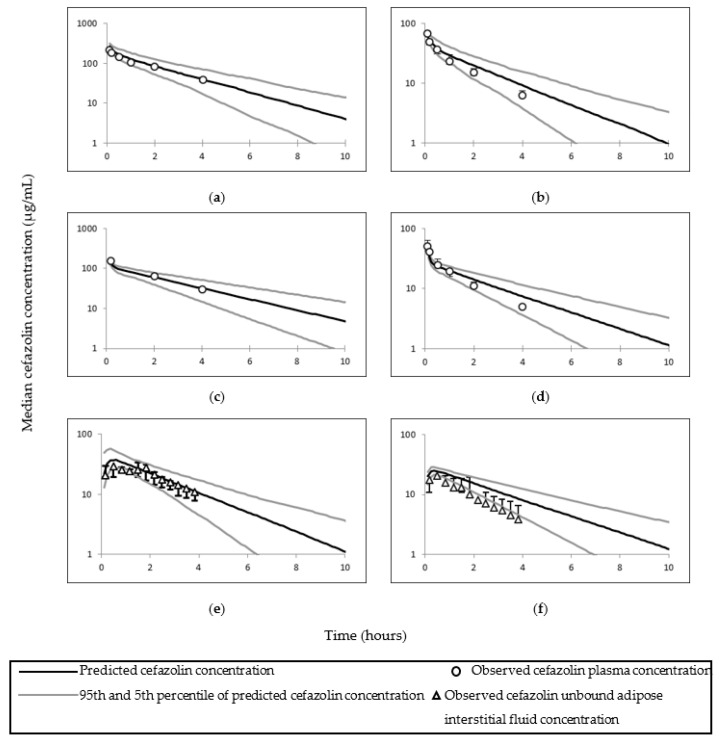
Simulated and observed cefazolin time concentrations curves in non–obese and obese subjects required surgical procedures. Time–concentration curves represent observed and simulated cefazolin (**a**) total plasma, (**b**) unbound plasma, and (**e**) unbound adipose tissue cefazolin concentrations in non–obese subjects; and simulated and observed (**c**) total plasma, (**d**) unbound plasma, and (**f**) unbound adipose tissue cefazolin concentrations in obese subjects [58].

**Figure 3:**
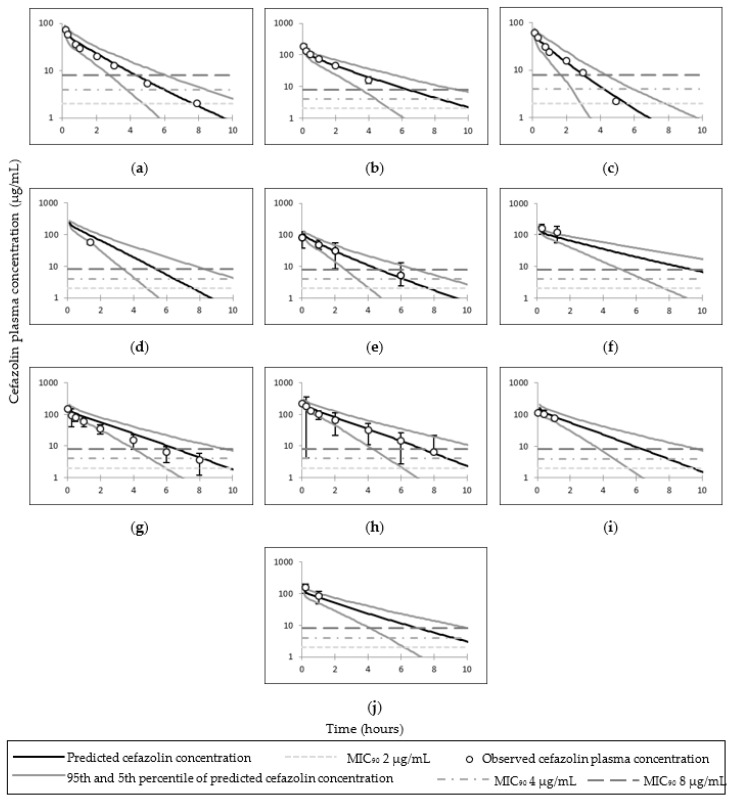
Simulated and observed plasma time–concentration curves of cefazolin in different populations. Plots (**a**) [8] and (**b**) [44] represent simulated and observed plasma time–concentration curves of cefazolin in lean non–pregnant subjects; (**c**) [8], (**d**) [2,45] and (**e**) [46] in pregnant subjects; (**f**) [47] in obese subjects; (**g**) [23], (**h**) [23], (**i**) [21] and (**j**) [22] in obese pregnant subjects. Error bars represent standard deviation in (**b**,**f**,**j**); interquartile range in (**d**,**i**); 95th and 5th percentile in (**e**); and standard error of mean in (**g**,**h**).

**Figure 4:**
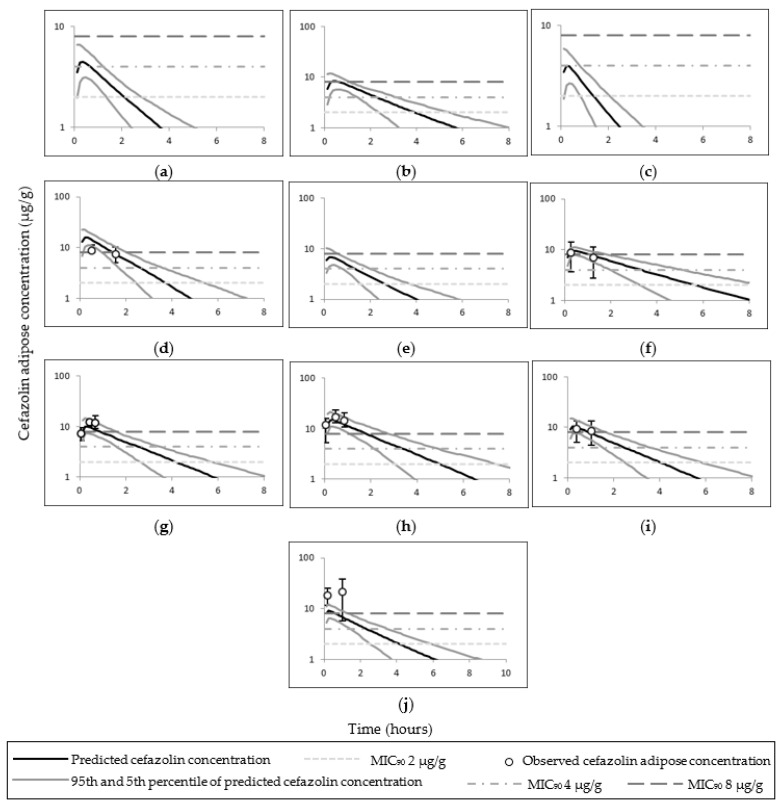
Simulated and observed adipose tissue time–concentration curves of cefazolin in different populations. Plots (**a**) [8] and (**b**) [44] represent simulated and observed adipose time–concentration curves of cefazolin in lean non–pregnant subjects; (**c**) [8], (**d**) [2,45] and (**e**) [46] in pregnant subjects; (**f**) [47] in obese subjects; (**g**) [23], (**h**) [23], (**i**) [21] and (**j**) [22] in obese pregnant subjects. Error bars represent interquartile range in (**d**,**g**–**i**); and standard deviations in (**f**,**j**).

The observed cefazolin total, free plasma, and adipose tissue ISF concentrations utilised to pilot cefazolin PBPK predictions and simulated concentrations are presented in Figure 2. Simulated cefazolin AUC and C_max_ (or the first reported time concentration point) were within two–fold of the observed data; the exception was the total adipose concentration in Stitely et al. (Table 4 and Table 5). The observed and mean simulated plasma concentrations time profiles for cefazolin are shown in Figure 3; and cefazolin total adipose concentrations time profiles are shown in Figure 4.

For one study, the simulated cefazolin total adipose concentrations in morbidly obese pregnant women (study code 0091), were not within the 2-fold of the clinically reported mean of 18.36 ± 6.68 µg/g at time of skin incision and 21.73 ± 16.02 µg/g at time of skin closure following 2000 mg cefazolin administration [22]. The simulated adipose concentration in lean pregnant at 31 min was 14.21 µg/g compared to an observed median value of 8.7 µg/g (interquartile range 5.7–11.2 µg/g); and at 82 min, the simulated adipose concentration was 8.3 µg/g compared to an observed value of 7.5 µg/g (interquartile range 5–10.1 µg/g) [2,45]. The simulated total adipose tissue cefazolin concentrations in obese–pregnant at 4, 24 25, 41 and 60 min were 5.29, 10.08, 9.85, 8.68 and 7.62 µg/g, respectively; compared to observed values of 7.4, 9.4, 12.4, 11.8 and 8.4 µg/g, respectively following 2000 mg cefazolin dose administrations (studies codes 007 and 009) [21,23].

All simulated cefazolin plasma concentrations (for the examined doses of 500, 1000, 2000 and 3000 mg) were above the MIC_90_, of 8 µg/mL for a mean time of 6.11 h, 4 µg/mL for a mean time of 7.92 h and 2 µg/mL for a mean time of 9.8 h following infusion. In an obese pregnant population (BMI ≥ 30 kg/m^2^), the simulated time of free cefazolin plasma concentrations were above the MIC_90_ (*f*T > MIC) of 8 µg/mL was a mean of 3.52 h, 4 µg/mL up to a mean of 5.28 h and 2 µg/mL up to a mean of 7.16 h following infusion of a 2000 mg dose (Supplementary materials, Results section Table S3). 

### 3.2. Cefuroxime PBPK Model

**Figure 5:**
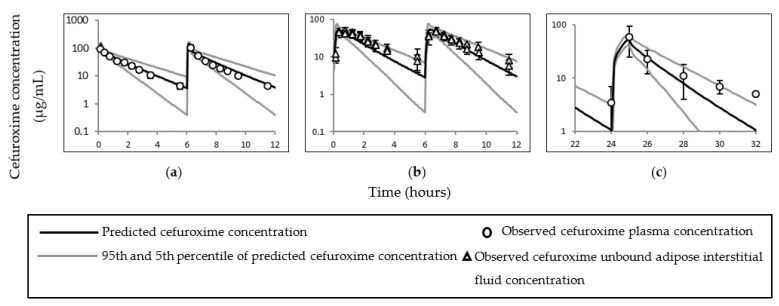
Simulated and observed cefuroxime time–concentration curves in subjects with different body mass index range. Time-concentration curves represent observed and simulated cefuroxime (**a**) total plasma and (**b**) adipose tissue cefuroxime concentrations in subjects (mean body mass index 25 kg/m^2^) required surgery [59], and (**c**) unbound plasma cefuroxime concentrations in subjects (mean ± SD body mass index 26.8 ± 4.5 kg/m^2^) required cerebral microdialysis for neurochemical monitoring [60].

The observed and simulated cefuroxime total, free plasma, and adipose tissue ISF concentrations utilised to preliminary predict cefuroxime concentrations are presented in Figure 5.

**Figure 6:**
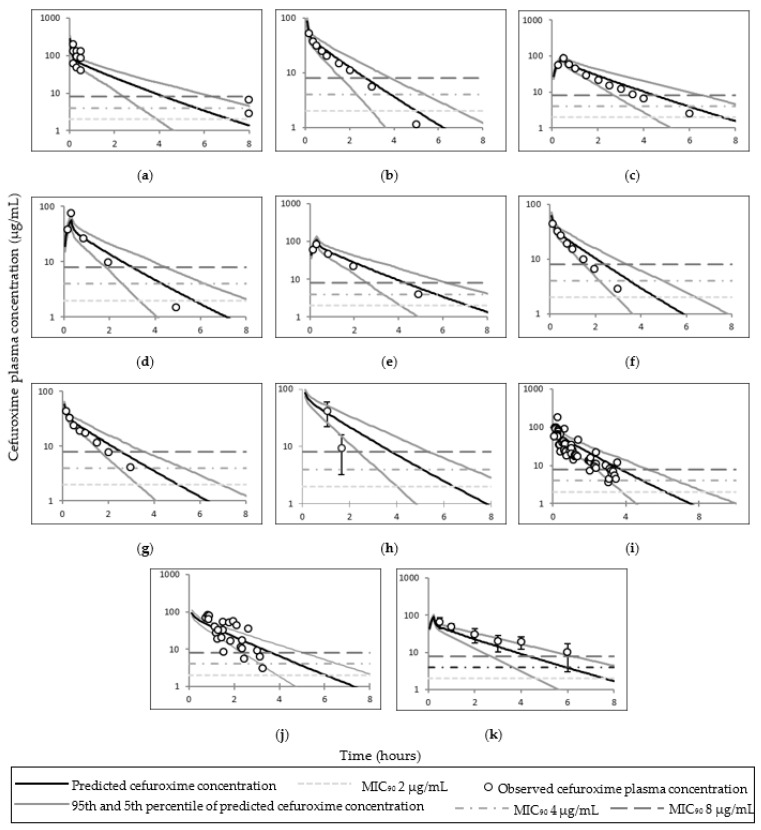
Simulated and observed plasma–time concentration curves of cefuroxime in different populations. Plots (**a**) [48], (**b**) [9], (**c**) [49], (**d**) [50] and (**e**) [50] represent simulated and observed plasma–time concentrations curves of cefuroxime in lean non–pregnant subjects; (**f**) [9], (**g**) [9], (**h**) [1], (**i**) [51] and (**j**) [51] in lean pregnant subjects; and (**k**) [52] in obese subjects. Error bars standard deviation of the observed concentrations in (**h**,**k**).

**Figure 7:**
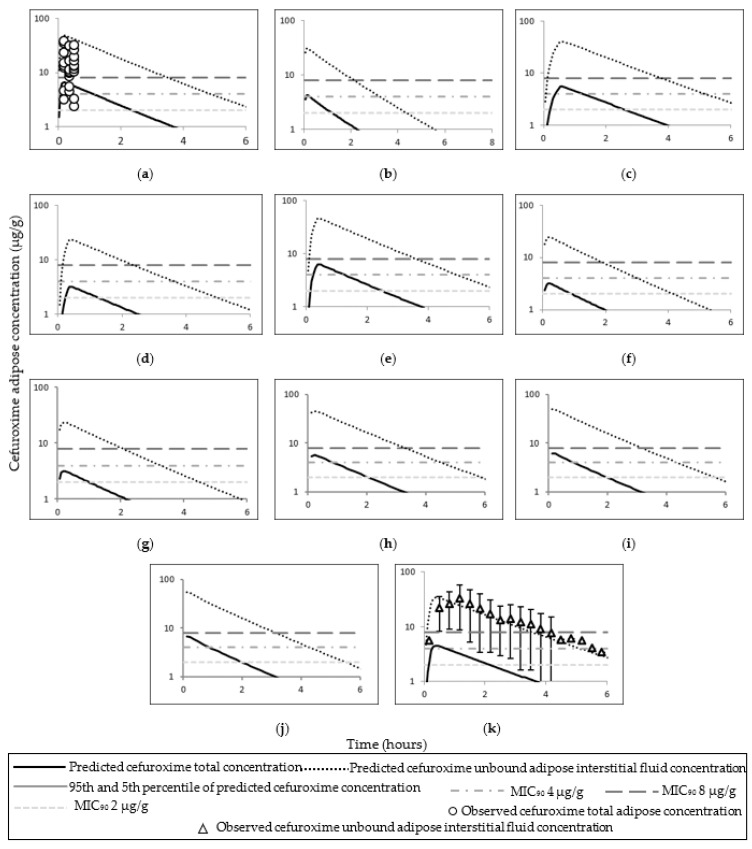
Simulated and observed adipose tissue–time concentration curves of cefuroxime in different populations. Plots (**a**) [48], (**b**) [9], (**c**) [49], (**d**) [50] and (**e**) [50] represent simulated and observed adipose–time concentrations curves of cefuroxime in lean non–pregnant subjects; (**f**) [9], (**g**) [9], (**h**) [1], (**i**) [51] and (**j**) [51] in pregnant subjects; (**k**) [52] in obese subjects. Error bars represent standard deviation of the observed concentrations in (**k**).

**Figure 8:**
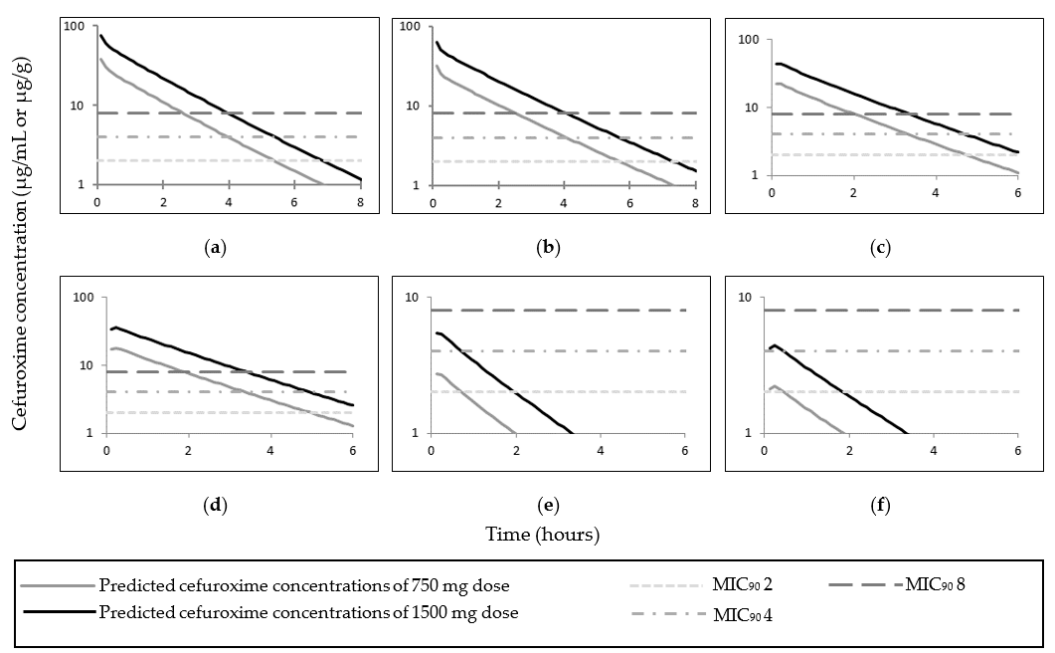
Simulated plasma, unbound adipose interstitial fluid, and total adipose time–concentrations curves of cefuroxime (750 and 1500 mg doses) in the obese pregnant population. Predicted mean cefuroxime plasma (**a**), unbound adipose interstitial fluid (**c**), and total adipose (**e**) concentrations in obese pregnant of 750 and 1500 mg doses; and predicted mean cefuroxime plasma (**b**), unbound adipose interstitial fluid (**d**), and total adipose (**f**) concentrations in morbidly obese pregnant of 750 and 1500 mg doses.

All simulated data of AUC and C_max_ (or first reported time concentration point) cefuroxime concentrations (serum and adipose tissue) were within 2–fold of the observed data with the exception of the adipose tissue concentrations in Lovering et al. (Table 6 and Table 7) (see Discussion Section 4.2 for more on this discrepancy). The observed and mean simulated plasma concentrations time profiles for cefuroxime are shown in Figure 6; and cefuroxime adipose concentrations time profiles are shown in Figure 7. Simulated cefuroxime plasma, unbound adipose ISF, and total adipose concentrations in the obese pregnant population are shown in Figure 8. The predicted cefuroxime plasma fifth percentile was superior in describing cefuroxime clearance phase compared to the predicted mean in lean pregnant subjects (Figure 6 f–j). The predicted fifth percentile of cefuroxime plasma concentration was utilised to evaluate cefuroxime T > MIC in both the lean and obese pregnant at time of CS. The predicted fifth percentile of cefuroxime plasma concentration in lean pregnant at term, was ≥MIC_90_ of 8 µg/mL up to 1.6 h post 750 mg cefuroxime dose; and 2.3 h post 1500 mg cefuroxime dose (Supplementary materials, Table S5).

### 3.3. Cefuroxime PBPK Model Application in the Obese Pregnant Population

Simulated 5th percentile cefuroxime plasma concentrations were above the MIC_90_ of 8 µg/mL for a duration of 1.63 h after 750 mg dose and 2.45 h after 1500 mg dose; and above 4 µg/mL for a duration of 2.45 h post 750 mg dose, and 3.45 h post 1500 mg dose and for both obese pregnant and morbidly obese pregnant populations (Supplementary materials Table S4). The simulated fifth percentile of cefuroxime *f*T > MIC of 8 µg/mL was 3.9 h, 4 µg/mL was 3 h, and 2 µg/mL was 2 h following 1500 mg dose in in both obese pregnant categories (studies code 2 and 4) (Supplementary materials Table S4). In all in silico scenarios, cefuroxime mean total adipose tissue concentrations were above the MIC_90_ of 2 µg/g, only higher doses of cefuroxime (i.e., 1500 mg) achieved an MIC_90_ ≥ 4 µg/g, the MIC_90_ of 8 µg/g was not reached in both obese pregnant categories. Cefuroxime unbound ISF concentrations were ≥8 µg/g for up to around 1.86 h (5th percentile: 1.25 h) after 750 mg dose and up to around 3.3 h (5th percentile: 2.2 h) after 1500 mg dose both obese pregnant and morbidly obese pregnant populations (Supplementary materials Table S5).

## 4. Discussion

The current study developed an obese pregnant PBPK model to describe plasma and adipose tissue concentrations of cefazolin and cefuroxime by integrating a lean pregnant PBPK model with known physiological covarites in non–pregnant obese subjects.

### 4.1. Cefazolin

The PBPK model of cefazolin was considered adequate in predicting both plasma and adipose concentrations of cefazolin. Predicted intravenous clearance and AUC were within two-fold of the observations and observed mean concentration profiles were within 5th–95th percentiles (Table 4 and Table 5 and Figure 3 and Figure 4). Additionally, higher dose of cefazolin (3000 mg) was adequately described via the PBPK obese pregnant model (study code 008) [23]. The under predicted values compared to observed values in Stitely et al. suggests higher distribution in vivo compared to the simulation.

The model indicates that cefazolin doses of ≥1000 mg can achieve a mean total cefazolin adipose tissue concentration above the MIC_90_ of 4 µg/g in all populations; while a dose of 500 can achieve similar MIC_90_ (i.e., 4 µg/g) in only lean non–pregnant subjects or lean pregnant subjects. A cefazolin dose of ≥2000 mg can achieve higher MIC_90_ (i.e., 8 µg/g) in all included populations. In all tested cefazolin scenarios, the unbound cefazolin adipose ISF concentrations were >8 µg/g. Increased body weight in pregnant women decreases concentrations (plasma and adipose) at a time point compared to lean pregnant, while the T > MIC are relatively similar or higher in obese compared to non–obese cohorts.

### 4.2. Cefuroxime

The PBPK cefuroxime model successfully predicted cefuroxime plasma, free plasma and ISF adipose concentrations. Limited data are available for cefuroxime exposure in homogenised total adipose tissue (both intracellular and extracellular) or intracellular solely. The model simulated comparable plasma concentration to the observed value at 60 min in pregnant women [1]. In a study of obese participants, where cefuroxime adipose tissue was sampled via clinical microdialysis to measure free ISF (extracellular) cefuroxime concentration, the simulated plasma and adipose concentrations were comparable to the observed values [52]. The differences between simulated and observed cefuroxime adipose tissue concentrations in Lovering et al. study possibly suggest higher in vivo intracellular cefuroxime penetration than of that predicted [48]. Cefuroxime doses of 750 mg and 1500 mg provided a free cefuroxime adipose ISF concentrations above the MIC_90_ of 8 µg/g for means of 2.07 h and 3.50 h, respectively, in lean non–pregnant, obese non–pregnant, and lean pregnant populations. Similar doses within different obese pregnant categorises had relatively comparable T > MIC and *f*T > MIC of plasma cefuroxime concentrations (Table S7).

In pregnant women at same gestational age with different body weight, although a comparable dose (1500 mg) achieved higher cefuroxime C_max_ (plasma or adipose) in lean-pregnant than obese cohorts, the simulated T > MIC were similar or shorter in lean pregnant compared to of those obese or morbidly obese pregnant (studies code 08–091, 2 and 4). This was also observed in predicted T > MIC of obese (non–pregnant) subjects compared to lean (non–pregnant) subjects (studies code 092 and 01). The elongated T > MIC of plasma, free plasma and ISF cefuroxime concentration with an increased weight can be explained by an increased cefuroxime half-life due to increased V_d_ in obese subjects, the effect of CL on drug disposition in obese subjects seems minor compared to V_d_ [61].

A prime factor affecting T > MIC after CS is timing of administration cefuroxime; longitudinal timing from cefuroxime administration to start of CS decreases T > MIC after surgery. It is recommended to administer prophylactic antibiotics 15–60 min before skin incision to allow drug distribution to adipose tissue and ensure sufficient T > MIC after completion of surgery [62]. This study emphasised timing from prophylactic antibiotic administration to skin incision; administration of 1500 mg cefuroxime 15, 30, or 60 min before skin incision simulated a plasma T > MIC of 8 µg/mL after skin incision in obese pregnant categories for the fifth percentile of 2.2, 1.95, and 1.45 h, respectively. And *f*T > MIC of 1.75, 1.5 and 1 h/s after virtual administration of 1500 mg at 15, 30, and 60 min before skin incision (Table S7). The current PBPK adequately predicted the mean time to reach maximum cefuroxime adipose tissue concentration (t_max_) in Hosmann et al. (free ISF) and Lovering et al. (total adipose), while the predicted free cefuroxime ISF adipose tissue concentrations t_max_ was shorter compared to observed time in Barbour et al. The simulated cefuroxime total adipose concentration t_max_ (1500 mg) in the obese pregnant was 7.2 min, and in the morbidly obese pregnant was 14.4 min. If a 750 mg cefuroxime dose was given 60 min pre-CS, at the end of 1 h duration CS (i.e., 2 h post cefuroxime dose), only around 45.2% of obese pregnant subjects will achieve a cefuroxime free plasma concentration ≥ 8 µg/mL; and 44.2% of unbound cefuroxime adipose tissue ISF. For the same scenarios (750 mg cefuroxime dose given virtually 60 min pre-CS), 37.5%of morbidly obese pregnant subjects will attain a cefuroxime free plasma concentration ≥ 8 µg/mL, and 36.2% will achieve a free cefuroxime ISF adipose tissue concentration of 8 µg/g or above at the end of 1 h duration CS. These data support cefuroxime administration of no longer than 30 min before skin incision in different obese pregnant categories to allow sufficient cefuroxime adipose T > MIC during and after surgery.

The reported mean cefuroxime total adipose tissue (intracellular and extracellular) penetration percentages compared to systematic plasma cefuroxime concentration was 16% post 1500 mg dose (19% at 30 min) [48]. The reported mean plasma concentrations of lean pregnant women undergoing CS at around 1 h post cefuroxime dosing were 14.9 µg/mL (750 mg dose) and 41.14 µg/mL (1500 mg dose) [1,26]. Direct extrapolation (of 19% cefuroxime penetrations to total adipose tissue compared to the predicted mean cefuroxime plasma concentrations) would suggest a total adipose concentration at around 1 h of 2.8 µg/g (750 mg) and 7.8 µg/g (1500 mg) in lean pregnant women. While such extrapolation in the obese or morbidly obese pregnant would propose total cefuroxime concentrations of around 4.5 µg/g (750 mg dose) and 8.9 µg/g (1500 mg dose) at 30 min post dosing according to the mean predicted plasma concentrations in such populations.

The current guidelines recommend re-dosing prophylactic antibiotic when the surgery duration exceeds the antibiotic half-life (time required to for C_max_ to decrease by 50%); the mean reported half-life of cefuroxime in pregnant women is 75.63 ± 22.10 min [1,25]. Other guidelines recommend re-dosing cefuroxime if surgery exceeds two half-lives of prophylactic antibiotic (i.e., redose if the CS is not completed after 2.5 h of cefuroxime administration) [63]. According to observed clinical cefuroxime plasma concentration in lean–pregnant and predicted mean and fifth percentile of cefuroxime plasma, free plasma and ISF adipose concentrations (irrespective to total cefuroxime adipose tissue concentration), cefuroxime 750 mg dose is sufficient if: (1) a MIC_90_ of 4 µg/g or µg/mL are desired for at least 95% of women at 1 h post dose (redose if the CS is delayed more than 2 h to maintain MIC_90_ ≥ 4 µg/g or µg/mL), or (2) a MIC_90_ of 8 µg/g or µg/mL are desired for at least 75% of women at 1 h post dose (consider redosing if the CS is delayed more than around 1.5 h post dose, to maintain cefuroxime plasma above target MIC_90_ of 8 µg/mL) (Tables S5 and S6). In other situations, cefuroxime 1500 mg dose is recommended if MIC_90_ of ≥ 8 µg/g are desired for at least 95% of women at 2 h post dose; if the CS is delayed > 2 h post cefuroxime administration of 1500 mg in lean, obese, or morbidly obese pregnant women, a second dose might be required to maintain cefuroxime concentrations ≥ 8 µg/g or µg/mL (Tables S5 and S6). Balancing the benefit vs. the maternal/foetal safety should be considered [64].

In the last decade, vast number of studies have reported antibiotic concentrations when given prophylactically at the time of clean–contaminated surgeries with inconsistent recommendations. Since 2011, eight trials were conducted to measure cefazolin concentrations in obese pregnant women undergoing CS, where all studies measured cefazolin plasma concentrations [2,19,20,21,22,23,45,65], six studies measured cefazolin total adipose tissue concentrations [2,20,21,22,23,45,65], and one study measured free cefazolin ISF adipose concentration [65].

The dilemma in prophylactic antibiotic dosing in different populations undergoing a procedure arises from a lack of MIC_90_ cut-off value standardisation and small sample size of the clinical studies. Another reason for disagreement within the literature regarding antibiotic posology in a surgical procedure is the imprecise guidelines of the targeted site or cellular targeted segment of antibiotic concentration; i.e., plasma, free plasma, total adipose (extracellular and intracellular) or/and adipose ISF (free extracellular) concentration.

Although this PBPK model adequately described unbound cefuroxime ISF adipose tissue concentrations obese subjects, a clinical study is required to measure cefuroxime adipose concentration at the site of skin incision in CS. If sub-therapeutic adipose tissue cefuroxime concentration is proven, timing and dosage of cefuroxime should be reviewed. If a target MIC_90_ is reached and sustained for sufficient time before the surgery interval, timing from cefuroxime administration to skin incision should be decreased (not less than 7 min) or the dose regimen augmented with a second dose rather than increasing a single dose of cefuroxime due to the time-killing properties of cefuroxime [66]. If the clinical dose of 1500 mg fails to attain the desired T > MIC or does not reach a desired MIC_90_ value at any point, a study is warranted to investigate the efficacy, safety, and transplacental transfer of higher cefuroxime doses or consider an alternative prophylactic antibiotic. A protocol has been developed by our group to investigate cefuroxime pharmacokinetics in obese pregnant women during CS, but the clinical study was postponed due to the COVID–19 pandemic [67].

The current study had a few limitations. Several studies used for the verification of pregnant and/or obese pregnant PBPK model were of women undergoing CS. In the current study, the models were not parametrised to count for the effect of CS on pharmacokinetics. It may be considered a limitation because fluid administered and the type of anaesthesia given at time of CS can alter the pharmacokinetics of drugs, mainly V_d_. Another possible limitation of the current prediction of adipose tissue antibiotic concentration that was based on the total subcutaneous adipose tissue in the body (i.e., not specifically in abdominal tissue). This study assumed that cefuroxime and cefazolin distributes evenly to different types of adipose tissues in the body. Additionally, changes in the activity of renal transporters during pregnancy, if modified by obesity, were not considered in the model for OAT1 and MRP4. The current PBPK model did not account for the reported cefazolin f_u_ dose-dependent kinetics; nevertheless, the model sufficiently described cefazolin concentrations post different escalating doses. Finally, no dynamic PD model was incorporated in the developed models.

## 5. Conclusions

The developed cefazolin and cefuroxime PBPK models for lean pregnant predicted plasma and adipose concentrations of cefazolin and cefuroxime in obese pregnant women adequately. A cefazolin dose of 2000 mg achieved cefazolin plasma concentrations ≥ 8 µg/mL for up to around 7 h. If a plasma MIC of ≥ 8 µg/mL is required for up to 2 h, a dose of 1500 mg is therapeutically superior compared to the 750 mg dose for obese pregnant women at time of CS. While this study presented the simulated data for adipose tissue cefuroxime concentrations in pregnant and obese pregnant women, a clinical study to quantify cefuroxime concentration in adipose tissue is necessary to validate the obese pregnant model and further investigate cefuroxime posology in the population of interest.

## Figures and Tables

**Table 1:** Physiochemical properties and pharmacokinetics values of cefazolin and cefuroxime for healthy subjects used in compounds building using Simcyp 20.

Compound	Cefazolin		Cefuroxime	
Parameter	Value	References	Value	References
Molecular weight (g/mol)	454.5	[38]	424.39	[6]
Log *p*	−0.58	[39]	−1.5 ^1^	
Compound Type	Monoprotic Acid		MonoproticAcid	[6]
Acid PKa	3.6	[39]	3.15	[6]
B/P	0.55	[40]	0.56	[13]
f_u_	0.225	[4,5]	0.67	[3]
V_ss_ (L/Kg)	0.1 ^2^		0.226 ^2^	
K_p_ Scalar	1		1	
Adipose permeability limited CL_PD_ L/h	0.1		0.1	
Lung tissue: plasma partition coefficient	0.2 ^3^	[41]	Default (0.43)	
Muscle tissue: plasma partition coefficient	0.03 ^3^	[41]	Default (0.25)	
Skin tissue: plasma partition coefficient	0.09	[42]	Default (0.53)	
Kidney tissue: plasma partition coefficient	Default (0.21)		Default (0.36)	
Cl_PD, basel_ (mL/min/10^6^ cells)	2.07 × 10^−8^	[40]	N/A	
Cl_PD, apical_ (mL/min/10^6^ cells)	2.07 × 10^−8^	[40]	N/A	
fu_Kidney cell_	1		N/A	
fu_Urine_	1		N/A	
CL_int,T_ (µL/min/10^6^ cells) by T_up,b_ (OAT1)	0.208	[40]	9.62 ^4^	[13]
CL_int,T_ (µL/min/10^6^ cells) by T_up,b_ (OAT3)	7.28	[40]	N/A	
CL_int,T_ (µL/min/10^6^ cells) by T_eff,a_ (MRP4)	41.43	[12]	10 ^5^	[13]

B/P blood to plasma partition ratio, Log *p* partition coefficient, f_u_ fraction unbound, V_ss_ volume of distribution at steady state, K_p_ tissue-to-plasma partition coefficient, CL_int,T_ in vitro transporter-mediated intrinsic clearance Cl_PD_ passive diffusion clearance, fu_Kidney cel__l_ fraction unbound in kidney cell, fu_Urine_ fraction unbound in urine, T_eff,a_ efflux transporter on apical membrane, T_up,b_ uptake transporter on basolateral membrane, N/A not applicable. ^1^ Measured using shake-flask method. ^2^ Predicted using Rodgers and Rowland method within the Simcyp simulator [30,43]. ^3^ Mean value from rats and rabbits [41]. ^4^ Optimised by Hsu et al. [13] using serum concentration time profile. ^5^ Optimised by Hsu et al. [13] based on urine data.

**Table 2:** Summary of study designs used for cefazolin physiologically based pharmacokinetic model building.

Population	Study Code and Reference	Population Model Used	Dose (mg)	Infusion Time (min)	Number of Subjects (Proportion of Female)	Age Years (Minimum-Maximum)	Weight (kg)	Mean GA (Weeks)
Lean non–pregnant	001 [8]	Sim-Healthy Volunteers	500	2	6 (1)	N/S ^1^	62.3	N/A
	002 [44]	Sim-Healthy Volunteers	1000	N/S (used 2 min)	17 (0)	21–42	N/S ^1^	N/A
Pregnant	003 [8]	Sim-Pregnancy	500	2	6 (1)	N/S ^1^	67.9 ^2^	24.5
	004 [2,45]	Sim-Pregnancy	2000	4	10 (1)	28 (23–32)	65.7 ^2^	39.1
	005 [46]	Sim-Pregnancy	1000	IV bolus (N/S used 1 min)	20 (1)	32.5 (23–43)	79.3 ^2^	39
Obese	006 [47]	Sim-Obese	2000	IV bolus (N/S used 1 min)	37 (0.7)	18–60	127 ^2^	N/A
Obese–pregnant	007 [23]	Sim-Pregnancy ^3^	2000	3	13 (1)	29 (23.5–34)	113.6 ^2,4^	39.3
	008 [23]	Sim-Pregnancy ^3^	3000	3	13 (1)	31(30–35)	108 ^2,5^	39
	009 [21]	Sim-Pregnancy ^3^	2000	IV bolus (N/S used 1 min)	28 (1)	30 (25.5–34)	103 ^2,6^	39.2
Morbidly obese–pregnant	0091 [22]	Sim-Pregnancy ^7^	2000	IV bolus (0.75 min)	11 (1)	31.09 ^1^	129.14 ^2^	38.75

GA gestational age, N/A not applicable, N/S not stated in the clinical study. ^1^ Used the default value of the selected population in Simcyp simulator. ^2^ Weight was adjusted within simulator to report the mean observed weight ± 5 Kg. ^3^ The pre-pregnancy tissue flow rate was modified as obese. ^4^ Reported BMI at time of delivery as 42.9 Kg/m^2^, suggested weight as 113.6 if height is around 163 cm. ^5^ Reported BMI at time of delivery as 41.8 Kg/m^2^, suggested weight as 108 if height is around 163 cm. ^6^ Reported BMI at time of delivery as 38.9 Kg/m^2^, suggested weight as 103 if height is around 163 cm. ^7^ The pre-pregnancy tissue flow rate was modified as morbidly obese.

**Table 3:** Summary of study designs used for cefuroxime physiologically based pharmacokinetic model building.

Population	Study Code and Reference	Population Model Used	Dose (mg)	Infusion (min)	Number of Subjects (Proportion of Female)	Age (Minimum–Maximum) Years	Weight (kg)	Mean GA (Weeks)
Lean non–pregnant	01 [48]	Sim-Healthy Volunteers	1500	N/S (used 2 min)	12 (N/S ^1^)	N/S ^1^	N/S ^1^	N/A
	02 [9]	Sim-Healthy Volunteers	750	1	7 (1)	N/S ^1^	61.7 ^2^	N/A
	03 [49]	Sim-Healthy Volunteers	1500	30	23 (0.26)	(19–31)	76 ^2^	N/A
	04 [50]	Sim-Healthy Volunteers	750	20	10 (0.5)	32 (18–48)	72	N/A
	05 [50]	Sim-Healthy Volunteers	1500	20	10 (0.5)	32 (18–48)	72 ^2^	N/A
Pregnant	06 [9]	Sim-Pregnancy	750	1	7 (1)	N/S ^1^	64.4	29
	07 [9]	Sim-Pregnancy	750	1	7 (1)	N/S ^1^	74	41 (used 40)
	08 [1]	Sim-Pregnancy	1500	1	18 (1)	23–37	78.94	39.5
	09 [51]	Sim-Pregnancy	1500	IV bolus (N/S used 1 min)	10 (1)	16–32 ^3^	74.4 ^2^	At term (used 39)
	091 [51]	Sim-Pregnancy	1500	IV bolus (N/S used 1 min)	10 (1)	17–36 ^3^	70.3 ^2^	At term (used 39)
Obese	092 [52]	Sim-Morbidly Obese	1500	15 ^4^	6 (1)	19–76	131.66 ^2^	N/A
Obese–pregnant	1	Sim-Pregnancy ^5^	750	1	50 (1)	23–37	97.8 ^6^	39.5
	2	Sim-Pregnancy ^5^	1500	1	50 (1)	23–37	97.8 ^6^	39.5
Morbidly obese–pregnant	3	Sim-Pregnancy ^7^	750	1	50 (1)	23–37	128 ^8^	39.5
	4	Sim-Pregnancy ^7^	1500	1	50 (1)	23–37	128 ^8^	39.5

GA gestational age, N/A not applicable, N/S not stated in the clinical study, PRG pregnant. ^1^ Used the default value of the selected population in Simcyp simulator. ^2^ Weight was adjusted within simulator to report the mean observed weight ± 5 Kg. ^3^ The minimal age for pregnant in Simcyp is 20 years. ^4^ Didn’t mention the exact infusion time, stated as “short term infusion” suggested as 15 min. ^5^ The pre-pregnancy tissue flow rate was modified as obese. ^6^ A pre-pregnancy weight of 80 Kg was selected. ^7^ The pre-pregnancy tissue flow rate was modified as morbidly obese. ^8^ A pre-pregnancy weight of 105 Kg was selected.

**Table 4:** Observed vs. simulated area under the curve, serum concentration and clearance of cefazolin.

Study Code and Reference	Dose (mg)	AUC (mg/L·h)			Cefazolin Serum Concentration (µg/mL) at Different Time Point				Clearance (L/h)		
		Obs.	Sim.	Ratio ^1^	Time Point (min)	Obs.	Sim. (5th, 95th Percentile)	Ratio ^1^	Obs.	Sim.	Ratio ^1^
001 [8]	500	110	130.27	1.18	C_10.8_	73.24	66.26 (51.82, 82.85)	0.90	4.04	3.91	0.97
002 [44]	1000	236.15 ^2^	262.60	1.11	C_4.4_	188.6	152.72 (128.60, 179.57)	0.81	3.8	4.01	1.06
003 [8]	500	75.7	84.37	1.11	C_9_	60.07	57.38 (45.56, 73.06)	0.96	7.3	6.22	0.85
004 [2,45]	2000	N/R ^3^	362.97	N/A	C_82_	57.2	95.74 (60.07, 135.05)	1.67	N/R	5.76	N/A
005 [46]	1000	139.27 ^2^	172.24	1.24	C_max_	82.53	105.60 (86.73, 126.67)	1.28	7.18	5.99	0.83
006 [47]	2000	N/R ^3^	415.86	N/A	C_16_	159.96	131.55 (110.07, 155.41)	0.82	N/R	5.04	N/A
007 [23]	2000	234.3	307.48	1.31	C_max_	146.15	163.31 (133.85, 203.67)	1.12	8.4	6.17	0.73
008 [23]	3000	453.4	446.82	0.99	C_max_	223.74	249.68 (199.69, 301.13)	1.12	6.6	6.35	0.96
009 [21]	2000	N/R ^3^	316.33	N/A	C_24.5_	100.7	116.01 (84.16, 161.83)	1.15	N/R	6.53	N/A
0091 [22]	2000	N/R ^3^	294.52	N/A	C_12_	155.45	115.28 (85.84, 153.94)	0.74	N/R	7.04	N/A

AUC area under the curve, C_max_ maximum concentration, N/A not applicable, N/R not reported, Obs. observed, Sim. simulated, numbers in brackets represent the 5th and 95th percentile. ^1^ Ratio of predicted/observed. ^2^ calculated using $\mathrm{Area}\;\mathrm{under}\;\mathrm{the}\;\mathrm{curve}=\frac{\mathrm{Dose}}{\;\mathrm{Clearance}\;}$. ^3^ The AUC could not be estimated, insufficient concentration–time points.

**Table 5:** Observed and simulated adipose tissue concentrations of cefazolin and simulated time above MIC_90_.

Study Code and Reference	Dose (mg)	Adipose Tissue Concentration (µg/g) at Time Point				Simulated Time (Hour) of Total Adipose Tissue Concentration above MIC_90_			Simulated Time (Hour) of Free ISF Adipose Tissue Concentration above MIC_90_		
		Time Point (min)	Obs.	Pred. (5th, 95th Percentile)	Ratio ^1^	T > MIC of 2 µg/g	T > MIC of 4 µg/g	T > MIC of 8 µg/g	T > MIC of 2 µg/g	T > MIC of 4 µg/g	T > MIC of 8 µg/g
001 [8]	500	C_max_	N/R	4.52 (3.12, 6.29)	N/A	2.04	0.6	NA	4.08	2.52	1.08
002 [44]	1000	C_max_	N/R	7.62 (5.17, 10.84)	N/A	3.6	2.04	NA	6	4.2	2.52
003 [8]	500	C_max_	N/R	4.01 (2.56, 5.72)	N/A	1.32	0.24	NA	3	1.8	0.72
004 [2,45]	2000	C_31.5_	8.7	14.21 (10.90, 18.55)	1.63	3.72	2.52	1.44	5.76	4.56	3.24
005 [46]	1000	C_max_	N/R	6.88 (4.47, 10.11)	N/A	2.52	1.32	NA	4.8	3.36	2.04
006 [47]	2000	C_16_	8.78	9.18 (7.33, 10.91)	1.05	5.52	3.24	0.96	9	6.48	4.08
007 [23]	2000	C_25_	12.4	9.85 (7.30, 13.86)	0.79	4.08	2.4	0.84	7.08	5.28	3.48
008 [23]	3000	C_28_	16.8	14.39 (10.78, 19.59)	0.86	5.04	3.36	1.8	8.04	6.24	4.44
009 [21]	2000	C_24.5_	9.4	10.08 (7.67, 13.79)	1.07	4.08	2.4	0.96	6.96	5.16	3.48
0091 [22]	2000	C_12_	18.36 ± 6.68	8.89 (6.16, 12.05)	0.48 ^2^	4.08	2.28	0.6	7.44	5.4	3.48
		C_60_	21.73 ± 16.02	6.85 (4.95, 9.01)	0.32 ^2^						

C_max_ maximum concentration, ISF interstitial fluid, N/A not applicable, N/R not reported, T > MIC Time above the minimum inhibitory concentration. ^1^ Ratio of predicted/observed. ^2^ Outside the 2–fold criteria (see Section 4).

**Table 6:** Observed vs. simulated area under the curve, serum concentration and clearance of cefuroxime.

Study Code and Reference	Dose(mg)	AUC (mg/L·h)			Cefuroxime Serum Concentration (µg/mL) at Time Point				Clearance (L/h)		
		Obs.	Sim.	Ratio ^1^	Time Point (min)	Obs.	Sim. (5th, 95th Percentile)	Ratio ^1^	Obs.	Sim.	Ratio ^1^
01 [48]	1500	N/R ^2^	150.99	N/A	C_10_	130.8	91.04 (69.23, 114.79)	0.70	N/R	11.36	N/A
02 [9]	750	60.8	75.79	1.25	C_9.6_	51.48	52.19 (41.15, 63.31)	1.01	11.9	12.14	1.02
03 [49]	1500	124	153.89	1.24	C_16.8_	56.35	65.21 (53.63, 78.74)	1.16	12.09 ^3^	10.99	0.91
04 [50]	750	77	78.03	1.01	C_20_	75.29	58.61 (45.73, 73.63)	0.78	9.74 ^3^	11.48	1.18
05 [50]	1500	137	157.20	1.15	C_18_	82.08	110.83 (86.74, 138.67)	1.35	10.9 ^3^	11.48	1.05
06 [9]	750	42.0	61.09	1.45	C_7.8_	43.56	45.29 (37.65, 53.94)	1.04	16.9	11.98	0.71
07 [9]	750	46.7	63.33	1.36	C_9_	43.83	39.73 (33.94, 46.55)	0.91	15.5	11.57	0.75
09 [51]	1500	N/R	121.51	N/A	C_30_	55.2	56.02 (42.21, 71.86)	1.01	N/R	11.92	N/A
091 [51]	1500	N/R	123.86	N/A	C_48_	74.8	45.51 (31.75, 57.04)	0.61	N/R	11.57	N/A
092 [52]	1500	158.7	130.76	0.82	C_30_	64.25	48.13 (39.02, 57.18)	0.75	8.39	12.33	1.47
Scenario 1	750	N/A	60.98	N/A	C_max_	N/A	37.75 (30.74, 45.99)	N/A	N/A	11.98	N/A
Scenario 2	1500	N/A	121.97	N/A	C_max_	N/A	75.51 (61.48, 91.97)	N/A	N/A	11.98	N/A
Scenario 3	750	N/A	57.40	N/A	C_max_	N/A	31.76 (25.84, 38.35)	N/A	N/A	12.85	N/A
Scenario 4	1500	N/A	114.79	N/A	C_max_	N/A	63.52 (51.67, 76.70)	N/A	N/A	12.85	N/A

AUC area under the curve, C_max_ maximum concentration, N/A not applicable, N/R not reported, Sim. simulated, numbers in brackets represent the 5th and 95th percentile. ^1^ Ratio of predicted/observed. ^2^ The AUC could not be estimated, insufficient concentration-time points. ^3^ Calculated using $\mathrm{Clearance}=\frac{\mathrm{Dose}}{\;\mathrm{Area}\;\mathrm{under}\;\mathrm{the}\;\mathrm{curve}\;}$.

**Table 7:** Observed and simulated adipose tissue concentrations of cefuroxime and simulated time above MIC_90_.

Study Code and Reference	Dose (mg)	Adipose Tissue Concentration (µg/g) at Time Point				Simulated Time (Hour) of Total Adipose Tissue Concentration above MIC_90_			Simulated Time (Hour) of Free ISF Adipose Tissue Concentration above MIC_90_		
		Time Point (min)	Obs.	Pred. (5th, 95th Percentile)	Ratio ^1^	T > MIC of 2 µg/g	T > MIC of 4 µg/g	T > MIC of 8 µg/g	T > MIC of 2 µg/g	T > MIC of 4 µg/g	T > MIC of 8 µg/g
01 [48]	1500	C_10_	16.5	6.55 (3.38, 10.48)	0.40 ^2^	2.35	1.09	N/A	6.32	4.88	3.49
02 [9]	750	C_max_	N/R	4.24 (2.44, 6.83)	N/A	1.14	0.18	N/A	4.32	3.18	2.10
03 [49]	1500	C_max_	N/R	5.53 (3.51, 7.93)	N/A	2.52	1.26	N/A	6.60	5.10	3.72
04 [50]	750	C_max_	N/R	3.19 (1.87, 4.60)	N/A	1.215	N/A	N/A	4.95	3.60	2.30
05 [50]	1500	C_max_	N/R	6.36 (3.84, 9.10)	N/A	2.46	1.20	N/A	6.36	4.92	3.60
06 [9]	750	C_max_	N/R	3.15 (2.25, 4.49)	N/A	0.9	N/A	N/A	4.14	3.00	1.86
07 [9]	750	C_max_	N/R	2.88 (2.16, 3.80)	N/A	0.84	NA	N/A	4.50	3.24	2.04
08 [1]	1500	C_max_	N/R	5.72 (4.35, 7.32)	N/A	2.04	0.84	N/A	5.76	4.44	3.24
09 [51]	1500	C_max_	N/R	6.14 (3.75, 10.81)	N/A	1.92	0.84	N/A	5.52	4.32	3.12
091 [51]	1500	C_max_	N/R	6.88 (3.86, 10.45)	N/A	1.92	0.84	NA	5.40	4.20	3.12
092 [52]	1500	C_max_	36.06 ^3^	33.74 ^3^ (29.62, 37.84)	0.94	2.1	0.66	N/A	6.72	5.10	3.48
Scenario 1 ^4^	750	C_max_	N/A	2.72 (1.82, 4.05)	N/A	0.6	N/A	N/A	4.68	3.24	1.92
Scenario 2 ^4^	1500	C_max_	N/A	5.43 (3.64, 8.10)	N/A	1.92	0.6	N/A	6.12	4.68	3.24
Scenario 3 ^4^	750	C_max_	N/A	2.22 (1.68, 2.88)	N/A	0.36	N/A	N/A	4.92	3.36	1.8
Scenario 4 ^4^	1500	C_max_	N/A	4.44 (3.36, 5.75)	N/A	1.8	0.36	N/A	6.48	4.92	3.36

C_max_ maximum concentration, N/A not applicable, N/R not reported, T > MIC time above the minimum inhibitory concentration, numbers in brackets represent the 5th and 95th percentile. ^1^ Ratio of predicted/observed. ^2^ Outside the 2-fold criteria. ^3^ Unbound adipose interstitial fluid. ^4^ Assessed scenarios: 750 mg (scenario 1) and 1500 mg (scenario 2) in the obese pregnant population and 750 mg (scenario 3) and 1500 mg (scenario 4) in the morbidly obese pregnant population.

## Data Availability

The data presented in this study are openly available in https://uquadmin-my.sharepoint.com/:f:/g/personal/hrammaal_uqu_edu_sa/Ep65sYVXVVJCiNqovDe7n-ABcYjz9Xhx86ddYcnbyTbosg (accessed on 23 March 2022).

## Supplementary Materials

The following supporting information can be downloaded at: https://www.mdpi.com/article/10.3390/pharmaceutics14061162/s1, Figure S1. Pregnancy (Maternal) PBPK model coupled with MechKiM model. Table S1. Tissue blood flow rates in female subjects. Table S2. Time above the minimum inhibitory concentration (2, 4 and 8 µg/mL) post a 2000 mg cefazolin doses given virtually to obese–pregnant and morbidly obese–pregnant. Table S3. Simulated time of cefazolin total and free plasma level above minimum inhibitory concentration required to inhibit the growth of 90% of organisms. Table S4. Simulated time of cefuroxime total and free plasma level above minimum inhibitory concentration required to inhibit the growth of 90% of organisms. Table S5. Time of simulated cefuroxime concentration (total plasma, free plasma, adipose tissue homogenate, and adipose ISF) above minimum inhibitory concentration required to inhibit the growth of 90% of organisms (2, 4 and 8 µg/mL or µg/g) post dose of 750 mg and 1500 mg, and dose efficacy when given cefuroxime 30 min or 60 min pre CS of 1 h. Table S6. Simulated percentages of obese– and morbidly obese–pregnant subjects achieving concentrations (total plasma, free plasma, adipose tissue homogenate, and adipose ISF) above minimum inhibitory concentration (2, 4 and 8 µg/mL or µg/g) at 1.5 h and 2 h post dose of 750 mg and 1500 mg. Table S7. Time above the minimum inhibitory concentration (2, 4 and 8 µg/mL) of different cefuroxime doses given virtually to obese–pregnant and morbidly obese–pregnant.
