# Supplementary material for: Application of a Physiologically Based Pharmacokinetic Model to Predict Cefazolin and Cefuroxime Disposition in Obese Pregnant Women Undergoing Caesarean Section
Source: Pharmaceutics. 2022 May 30;14(6):1162. doi: 10.3390/pharmaceutics14061162 (PMC9229966; doi:10.3390/pharmaceutics14061162)
Supplement: Supplementary file 1 [file pharmaceutics-14-01162-s001.zip › pharmaceutics-1726304-supplementary.pdf]

pharmaceutics

Supplementary Materials: Application of a Physiologically Based Pharmacokinetic Model to Predict Cefazolin and Cefuroxime Disposition in Obese Pregnant Women Undergoing Caesarean Section

Hanadi H. Alrammaal, Khaled Abduljalil, Victoria Hodgetts Morton, R. Katie Morris, John F. Marriott, Hsu P. Chong and Hannah K. Batchelor

S1. Method

S1.1. Study selection for the Physiologically-based Pharmacokinetics models:

The selection of studies used in cefazolin or cefuroxime Physiologically-based Pharmacokinetics (PBPK) models was dependent on the availability of observed cefazolin and cefuroxime adipose tissue concentrations.

S1.1.1. Cefazolin

The Philipson study [1] was selected because it reported cefazolin PK in healthy non-obese women during and after pregnancy.

Ritta and Ravin used different dosing than Philipson et al. [1,2]. Elkomy et al. represented cefazolin PK at the time of caesarean section (CS) compared to other studies at the time of pregnancy [3]. Pevzner et al. reported cefazolin concentrations in adipose tissue in normal-weight pregnant women at the time of CS [4,5]. Chen et al. reported cefazolin concentrations in adipose tissue in obese subjects undergoing bariatric surgery with no other conditions [6].

Many studies analysed cefazolin in the obese-pregnant population [4,7-12]. Out of 40 score, the highest ClinPK scores were reported by Stitely et al. (39), Young et al. (38), Kram et al. (37) and Maggio et al. (36) [8,9,11,12]. Kram et al. used a modified Kirby-Baure assay, which is not considered to be a robust technique for analysing cefazolin in blood and adipose tissue. As a result, Young et al., Maggio et al. and Stitely et al. were selected to investigate the appropriately of the PBPK obese-pregnant model to predict cefazolin concentrations in plasma and adipose compared to observed data.

S1.1.2. Cefuroxime

The study by Lovering et al. (lean non–pregnant population) was selected because it reported cefuroxime adipose tissue concentrations in subjects underwent hip replacement [13]. Studies by Kegdel et al. and Garton et al. (healthy populations) were used previously in the study by Hsu et al. to predict cefuroxime concentrations in pregnancy PBPK model using Simcyp V12.1 [14-16]. These studies were included in this work to ensure reproducibility of in-silico prediction of Simcyp version 20 to the older version.

For the Philipson and Stiernstedt study, healthy pregnant women of normal weight were selected as representative of the gestational differences in women during pregnancy and at delivery [17]. Additionally, it identified the PK of cefuroxime of these women after delivery, on the return of the menstrual cycle and cessation of breastfeeding. This data will allow the comparison of the cefuroxime PK parameter during pregnancy with the healthy population. Bousfield et al. investigated cefuroxime concentrations in pregnant women at time whom required labour induction or CS [18]. Lalic et al. was selected because they reported the highest Clin PK score among other related studies [19,20]. Barbour

et al. reported cefuroxime concentrations in the blood and adipose tissue of obese women undergoing abdominal surgery [21].

S1.2. Cefuroxime logP

The Log P used in Hsu et al. was -0.9; this was predicted using ADMET Predictor™ [16]. In our study, Log P was measured using the shake-flask method. Cefuroxime was dissolved in 100 ml of water (200 µg/ml); 3 ml of this solution was added to 3 ml of n-octanol and that solution was shaken for 24 hours at 4°C. Then, the two phases were allowed to separate for 24 hours and after separation the cefuroxime content was measured by high-performance liquid chromatography in both the water and the n-octanol using a previously published validated method with minor modification [12,21]. The partition coefficient of the cefuroxime was calculated using equation (S1)[22]. The resulting Log P was -1.5. A sensitivity analysis of cefuroxime Log P range values from -3 to 3 was performed using cefuroxime-observed plasma concentrations in healthy subjects.

$$\mathrm{LogP_{oct/wat}} = \mathrm{Log}\left(\frac{[\text{solute in octanol}]}{[\text{solute in water}]}\right) \quad \textbf{(S1)}$$

S1.3. PBPK models of non–obese non–pregnant, obese, pregnant and obese–pregnant populations

Figure S1 represents the pregnancy PBPK model coupled with the generic permeability-limited adipose and mechanistic kidney permeability model used within the Simulator [23-26]. For both cefazolin and cefuroxime, the model was firstly established for the non–obese and non–pregnant population. This is the structural or "baseline" model. Then changes in physiology during pregnancy were added in the model by selecting the Sim-pregnancy population within the Simulator [27]. In a parallel step, the physiological changes to obese populations were added to the model by selecting the non–pregnant Sim-Obese and Sim-Morbidity Obese populations within the Simulator [28] to verify the model adequacy of describing any change in the PK in these population. The last step was to incorporate the changes due to obesity in the baseline model for pregnancy model. In all cases the compound-specific parameters (model input table 1 in the main text in the manuscript) were remain without any modification across the different populations. For obese–pregnant PBPK model, in addition to the bodyweight adjustment, the tissue blood flows of obese and morbidly obese subjects were used as the baseline (pre-pregnant) of obese– and morbidly obese–pregnant PBPK model (Table S1).

S1.3.1. Organs, tissue and blood flow

Tissue volume

The maternal pregnancy model uses a lumped feto-placental compartment to account for the growth and change in the fetal, placental, uterine and amniotic fluid during pregnancy. Gestational age-dependent equations describing changes to the fetoplacental compartment and other tissue volumes, such as plasma volume, red blood cell volume and total fat mass as follows: are described (S2), (S3), (S4) and (S5), respectively.

$$\text{Fetoplacental volume} = 0.08\ (1 - 0.245\ \mathrm{GA} + 0.05375\ \mathrm{GA}^2\ ) \quad \text{(S2)}$$

$$\text{Plasma volume, PV, (L)} = \mathrm{PV_0}\ (1 + -0.00892\ \mathrm{GA}\ +\ 0.00168\ \mathrm{GA}^2\ -\ 0.000028\ \mathrm{GA}^3) \quad \text{(S3)}$$

$$\text{Red Blood Cell volume, RBCV, (L)} = \mathrm{RBCV_0} * (1\ +\ 0.00658\ \mathrm{GA}) \quad \text{(S4)}$$

$$\text{Adiopose Volume, AdipV, (L)} = \text{AdipV}_0\ (1 \pm\ 0.004168\ \text{GA} +\ 0.000743\ \text{GA}^2 - 0.000012\ \text{GA}^3) \quad \text{(S5)}$$

Where PV_0, $RBCV_0$, $AdipV_0$ represent the baseline in non-pregnant female population, predicted within the Simulator from the underlying covariates [29]. GA is Gestational age in weeks

Blood flows

The change in cardiac output and consequent alteration of blood flow percentages to the kidneys, brain, adipose tissues, and the new organ (feto-placenta) are incorporated into the Sim–pregnancy model. Equations (S6), (S7), (S8), (S9) and (S10) represent the gestational changes of cardiac output and blood flows to the uterine (and its contents, i.e. feto-placental compartment), kidneys (effective renal blood flow), adipose and skin, respectively [24].

$$\text{Cardiac output (L/h)} = \text{CO}_0\ (1 +\ 0.019657\ \text{GA} -\ 0.000292\ \text{GA}^2) \quad \textbf{(S6)}$$

$$\text{Fetoplacenta blood flow} = \text{FPBF}_0\ (1 + 0.070667\ \text{GA} + 0.034667\ \text{GA}^2 - 0.00067\ \text{GA}^3) \quad \textbf{(S7)}$$

$$\text{Renal blood flow } (\%CO) = \text{RBF}_0(1 + 0.024453\ \text{GA}\ -\ 0.00076\ \text{GA}^2) \quad \textbf{(S8)}$$

$$\text{Adipose blood flow, ABF, (\%CO)} = \text{ABF}_0\ (1 - 0.003412\ \text{GA} + 0.000059\ \text{GA}^2) \quad \textbf{(S9)}$$

$$\text{Skin blood flow (\%CO)} = \text{SBF}_0(1 + 0.0075\ \text{GA} + 0.0002\ \text{GA}^2) \quad \textbf{(S10)}$$

Where CO_0, $FPBF_0$, RBF_0, SBF_0, and ABF_0 are the baseline values for the non-pregnant female population; GA is Gestational age in weeks

Blood binding

Both cefazolin and cefuroxime are bound to albumin in plasma. The gestational-dependent change in plasma albumin in the model was accounted for according to the following equation.

$$\text{Albumin (g/L)} = \text{Albumin}_0\ (1\ -\ 0.00388\ \text{GA} -\ 0.000072\ \text{GA}^2) \quad \textbf{(S11)}$$

Where $Albumin_0$ is the baseline value for the non-pregnant female population predicted within the Simulator; GA is Gestational age in weeks

Renal function

The glomerular filtration rate value in non-pregnant population (GFR_0) is simulated using the modification of diet in renal disease (MDRD) equation (referred to as Method 2 in Simcyp®); equation (S12) represent this method [28,30]. The complex gestational changes of renal function have been described algorithmically by Abduljalil et al. [24]. The key physiological element of renal function is the GFR; the gestational alteration of GFR is described in equation (S13) [24].

$$\text{GFR}_0\ = 175 * (\frac{serum\ Creatine}{88.42})^{-1.154} * (age)^{-0.203}\ \ [* \ 0.742\ if\ Female] \quad \textbf{(S12)}$$

$$\text{Glomerular Filtration Rate, GFR, (ml/min/1.73}m2) =\ \text{GFR}_0(1 +\ 0.028392\ \text{GA} -\ 0.000502\ \text{GA}^2) \quad \textbf{(S13)}$$

Where GFR_0 is the baseline glomerular filtration rate (mL/min/1.73m2) values for the non-pregnant female population predicted within the Simulator using the equation (S12); GA is Gestational age in weeks.

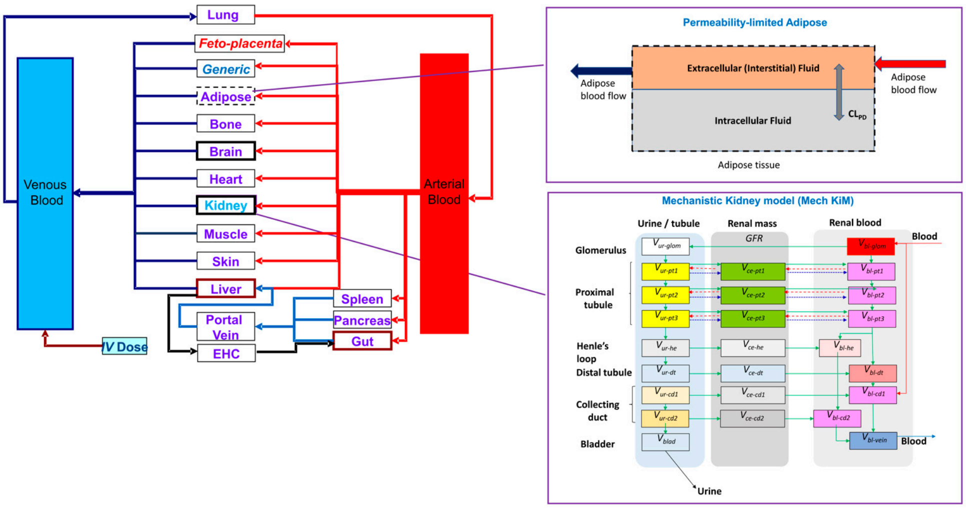

Figure S1. Pregnancy (Maternal) PBPK model coupled with MechKiM model.Note: For MechKiM model: both renal blood flow and GFR are gestational age dependent, hence the flows between kidney compartments are gestational age dependent (Solid black arrows). Dashed arrows represent directions of efflux and uptake transporters.

Table S1. Tissue blood flow rates in female subjects.

	Predefined tissue blood flow as percentage of cardiac output (Mean predicted L/h)			Mean predicted tissue blood flow L/h using discussed equations at 40 weeks gestational		
**Population**	**Non–obese, non–pregnant (baseline of pregnant population)**	**Obese (baseline of obese–pregnant population)**	**Morbidly obese (baseline of morbidity obese–pregnant population)**	Lean– pregnant	obese–pregnant	Morbidly obese–pregnant
**Adipose**	8.5 (20.62)	14.8 (37.34)	17.2 (58.42)	29.34	62.07	80.01
**Bone**	5 (17.42)	5 (17.36)	5 (18.63)	13.66	16.59	18.41
**Brain**	12 (41.81)	10.4 (37.36)	9.7 (36.50)	39.02	41.09	42.51
**Stomach and esophagus**	1 (3.48)	0.9 (3.12)	0.8 (2.98)	2.73	2.99	2.95
**Small intestine**	11 (35.75)	9.5 (31.98)	8.9 (32.42)	30.05	31.53	32.76
**Villi**	6 (20.90)	5.4 (18.56)	4.9 (18.38)	16.39	17.26	18.04
**Large intestine**	5 (14.85)	4.3 (13.17)	4 (14.04)	13.66	14.27	14.73
**Heart**	5 (14.85)	5 (14.85)	5 (17.40)	13.66	16.59	18.41
**Kidney**	17 (64.36)	14.7 (57.56)	13.8 (53.87)	46.68	49.05	51.07
**Liver (arterial)**	6.5 (22.64)	5.6 (20.20)	5.3 (19.87)	17.76	18.59	19.51
**Liver (portal)**	21.5 (68.48)	18.5 (61.22)	17.4 (62.85)	58.73	61.40	64.05
**Lung**	100 (348.38)	100 (347.21)	100 (372.52)	360.33	437.79	485.60
**Muscle**	12 (54.65)	12 (54.21)	12 (50.85)	32.78	39.83	44.18
**Pancreas**	1 (3.48)	1 (3.47)	1 (3.73)	2.73	3.32	3.68
**Skin**	5 (17.42)	6.6 (21.41)	7.2 (26.45)	29.19	46.81	56.64

**Spleen**	3 (7.88)	2.5 (7.02)	2.4 (8.08)	8.19	8.63	8.84
**Feto-Placenta**	0.6			35.49	43.12	47.82

S2. Results

Table S2 represent predicted T>MIC and *f*T>MIC of a 2000 mg cefazolin dose when given 15, 30 and 60 min before skin incision in obese– and morbidly obese–pregnant women.

Tables S3 and S4 represent simulated time above the minimum inhibitory concentrations required to inhibit the growth of 90% of most common organisms causing post CS infection (MIC_{90}) of cefazolin and cefuroxime, respectively.

Predicted time above MIC_{90} (2, 4 and 8 μg/mL or μg/g) of cefuroxime plasma, free plasma and adipose tissue concentration in lean non–pregnant, lean–pregnant, obese– and morbidly obese–pregnant after administration of either 750 mg or 1500 mg cefuroxime dose are presented in table S5. The percentages of subjects attaining a target MIC_{90} at 1.5 hours and 2 hours are illustrated in table S6. The 1.5 hours and 2 hours were selected to investigate percentages of subjects achieving targeted MIC post cefuroxime administration of 30 min or 60 min pre CS of 1 hours.

Table S2. Time above the minimum inhibitory concentration (2, 4 and 8 μg/mL) post a 2000 mg cefazolin doses given virtually to obese–pregnant and morbidly obese–pregnant.

Time from administration to skin incision	MIC_{90} (μg/mL)	Obese–pregnant women		Morbidly obese–pregnant	
		T>MIC (hours)	*f*T>MIC (hours)	T>MIC (hours)	*f*T>MIC (hours)
15 min	2	10.31 (5.63, 14.51)	6.83 (3.95, 9.83)	11.03 (5.87, 15.47)	7.19 (4.19, 10.31)
	4	8.39 (4.67, 12.11)	5.03 (2.99, 7.19)	8.87 (4.91, 12.59)	5.15 (2.99, 7.43)
	8	6.47 (3.71, 9.35)	3.35 (1.91, 4.79)	6.83 (3.83, 9.83)	3.23 (1.79, 4.91)
30 min	2	10.06 (5.38, 14.26)	6.58 (3.7, 9.58)	10.78 (5.62, 15.22)	6.94 (3.94, 10.06)
	4	8.14 (4.42, 11.86)	4.78 (2.74, 6.94)	8.62 (4.66, 12.34)	4.9 (2.74, 7.18)
	8	6.22 (3.46, 9.1)	3.1 (1.66, 4.54)	6.58 (3.58, 9.58)	2.98 (1.54, 4.66)
1 h	2	9.56 (4.88, 13.76)	6.08 (3.2, 9.08)	10.28 (5.12, 14.72)	6.44 (3.44, 9.56)
	4	7.64 (3.92, 11.36)	4.28 (2.24, 6.44)	8.12 (4.16, 11.84)	4.4 (2.24, 6.68)
	8	5.72 (2.96, 8.6)	2.6 (1.16, 4.04)	6.08 (3.08, 9.08)	2.48 (1.04, 4.16)

MIC_{90} minimum inhibitory concentration required to inhibit the growth of 90% of organisms, *f*T>MIC time of free concentration above the minimum inhibitory concentration, T>MIC Time above the minimum inhibitory concentration. Numbers in brackets represent T>MIC and , *f*T>MIC of 5th, and 95th percentile.

Table S3. Simulated time of cefazolin total and free plasma level above minimum inhibitory concentration required to inhibit the growth of 90% of organisms.

**Study code**	**Dose (mg)**	**Time (hour) for simulated cefazolin total plasma concentration above MIC_{90}**			**Time (hour) for simulated free plasma cefazolin concentration above MIC_{90}**		
		T>MIC of 2 μg/mL	T>MIC of 4 μg/mL	T>MIC of 8 μg/mL	*f*T>MIC of 2 μg/mL	*f*T>MIC of 4 μg/mL	*f*T>MIC of 8 μg/mL
**001 *[1]***	500	7.68	5.88	4.32	4.08	2.52	1.08
**002 *[2]***	1000	10.2	8.16	6.24	5.88	4.08	2.4
**003 *[1]***	500	5.4	4.2	2.88	2.88	1.8	0.72
**004 *[4,5]***	2000	8.4	6.96	5.64	5.88	4.56	3.24
**005 *[3]***	1000	7.68	6.12	4.56	4.8	3.36	2.04
**006 *[6]***	2000	14.76	12	9.36	9.12	6.48	4.08
**007 *[9]***	2000	10.56	8.64	6.72	7.08	5.28	3.6
**008 *[9]***	3000	11.64	9.6	7.68	8.04	6.24	4.44
**009 *[11]***	2000	10.44	8.52	6.6	6.96	5.16	3.48
**0091 *[8]***	2000	11.28	9.12	7.08	7.44	5.4	3.48

**Mean in all population (001-0091)**	All doses	9.80	7.92	6.11	6.22	4.49	2.86
	Only 2000	11.09	9.05	7.08	7.30	5.38	3.58
**Mean in Obese-pregnant (007, 009 and 0091)**	2000	10.76	8.76	6.80	7.16	5.28	3.52

fT>MIC time of free concentration above the minimum inhibitory concentration, T>MIC Time above the minimum inhibitory concentration

Table S1.Simulated time of cefuroxime total and free plasma level above minimum inhibitory concentration required to inhibit the growth of 90% of organisms

**Study code**	**Dose (mg)**	**Time (hour) for the simulated cefuroxime total plasma concentration above MIC$_{90}$**			**Time (hour) for si[illegible] ime concentration [illegible]**	
		T>MIC of 2 µg/mL	T>MIC of 4 µg/mL	T>MIC of 8 µg/mL	*f*T>MIC of 2 µg/mL	*f*T[illegible] µ[illegible]
**01 [13]**	1500	7.15	5.65	4.21	6.24	4.[illegible]
**02 [17]**	750	4.98	3.78	2.70	4.32	3.[illegible]
**03 [14]**	1500	7.44	5.88	4.44	6.54	5.[illegible]
**04 [15]**	750	5.67	4.28	2.97	4.86	3.[illegible]
**05 [15]**	1500	7.14	5.70	4.26	6.30	4.[illegible]
**06 [17]**	750	4.68	3.48	2.34	4.08	2.[illegible]
**07 [17]**	750	5.04 (3.2, 6.9)	3.72(2.4, 5.3)	2.52(1.6, 3.5)	4.44(2.9, 6.1)	3.[illegible]
**08 [19]**	1500	6.36 (4, 8.9)	5.04(3.1, 7.1)	3.72(2.3, 5.3)	5.76(3.6, 7.9)	4.[illegible]
**09 [18]**	1500	6.12(3.72, 8.28)	4.80(3, 6.72)	3.60(2.28, 5.16)	5.52(3.48, 7.56)	4.[illegible]
**091 [18]**	1500	6 (3.84, 8.04)	4.8 (3.12, 6.60)	3.6 (2.28, 5.04)	5.4 (3.60, 7.44)	4.[illegible]
**092 [21]**	1500	7.56	5.88	4.26	6.66	5.[illegible]
**1**	750	5.28(3.4, 7.8)	3.96(2.4, 6)	2.52 (1.7, 4)	4.68(3, 7)	3.[illegible]
**2**	1500	6.84(4.2, 9.6)	5.28(3.4, 7.8)	3.96(2.4, 5.9)	6.12(3.8, 8.9)	4.[illegible]
**3**	750	5.64(3.5, 8.3)	4.08(2.5, 6)	2.52(1.56, 3.8)	4.92(3, 7.3)	3.[illegible]
**4**	1500	7.32 (4.4, 10.6)	5.64 (3.5, 8.3)	4.08 (2.5, 6)	6.6 (4, 9.5)	4.[illegible]
**Mean of studies 01-05 and 092**	Both 750 and 1500	6.66	5.19	3.81	5.82	4.[illegible]
**Mean of studies 01-091**	Both 750 and 1500	6.19	4.82	3.51	5.47	4.[illegible]
**Mean in all population (01-4)**	Both 750 and 1500	6.21	4.80	3.45	5.50	4.[illegible]
**Mean of obese-pregnant population BMI≥ 30 kg/m^2 (1-4)**	Both 750 and 1500	6.27	4.74	3.27	5.58	4.[illegible]
**Mean of obese-pregnant population BMI≥ 30 kg/m^2 (2 and 4)**	1500	7.08	5.46	4.02	6.36	4.[illegible]
**The 5th percentile of obese-pregnant population BMI≥ 30 kg/m^2 (1-4)**	Both 750 and 1500	3.87	2.95	2.04	3.45	2.[illegible]
**The 5th percentile of obese-pregnant population BMI≥ 30 kg/m^2 (1 and 3)**	Only 750	3.45	2.45	1.63	3	2
**The 5th percentile of obese-pregnant population BMI≥ 30 kg/m^2 (2 and 4)**	Only 1500	4.3	3.45	2.45	3.9	3

*f*T>MIC time of free concentration above the minimum inhibitory concentration, T>MIC Time above the minimum inhibitory concentration

Table S2. Time of simulated cefuroxime concentration (total plasma, free plasma, adipose tissue homogenate, and adipose ISF) above minimum inhibitory concentration required to inhibit the growth of 90% of organisms (2, 4 and 8 μg/mL or μg/g) post dose of 750 mg and 1500 mg, and dose efficacy when given cefuroxime 30 min or 60 min pre CS of 1 hour.

population	MIC_{90}	Simulated mean T>MIC_{90} (hours) of 750 mg cefuroxime dose						Simulated mean T>MIC (hours) of 1500 mg cefuroxime dose					
		Plasma		Adipose		Dose recommendation if cefuroxime was given 30 or 60 min before surgery (of 1 hour duration CS)		Plasma		Adipose		Dose recommendation if cefuroxime was given 30 or 60 min before surgery (of 1 hour duration CS)	
		Total	Free	Total (Tissue homogenate)	ISF	30 min or less	60 min	Total	Free	Total (Tissue homogenate)	ISF	30 min or less	60 min
Lean Non–pregnant	2	4.98	4.32	1.14	4.32	Sufficient	Sufficient	7.15	6.24	2.35	6.32	Sufficient	Sufficient
	4	3.78	3.12	0.18	3.18	Sufficient	Sufficient	5.65	4.80	1.09	4.88	Sufficient	Sufficient
	8	2.70	2.04	N/A	2.10	Sufficient	Sufficient	4.21	3.36	N/A	3.49	Sufficient	Sufficient
Lean–Pregnant	2	5.04 (3.2, 6.9)	4.44 (2.9, 6.1)	0.84	4.5 (3, 6.1)	Sufficient	Sufficient	6.36 (4, 8.9)	5.76 (3.6, 7.9)	2.04	5.76 (3.7, 8)	Sufficient	Sufficient
	4	3.72 (2.4, 5.3)	3.18 (2.1, 4.4)	N/A	3.24 (2.2, 4.5)	Sufficient	Sufficient	5.04 (3.1, 7.1)	4.44 (2.8, 6.2)	0.84	4.44 (2.9, 6.2)	Sufficient	Sufficient
	8	2.52 (1.6, 3.5)	1.98 (1.3, 2.8)	N/A	2.04 (1.4, 2.9)	Sufficient	Sufficient	3.72 (2.3, 5.3)	3.24 (1.9, 4.6)	N/A	3.24 (2.2, 4.6)	Sufficient	Sufficient
Obese–pregnant	2	5.3 (3.4, 7.8)	4.7 (3, 7)	0.6	4.68 (3, 6.8)	Sufficient	Sufficient	6.84 (4.2, 9.6)	6.12 (3.8, 8.9)	1.92	6.12 (3.8, 8.8)	Sufficient	Sufficient
	4	3.96 (2.4, 6)	3.36 (2, 5)	N/A	3.24 (2.2, 4.9)	Sufficient	Sufficient	5.28 (3.4, 7.8)	4.68 (3, 7)	0.6	4.68 (3, 6.8)	Sufficient	Sufficient
	8	2.52 (1.7, 4)	**1.92 (1.2, 3)**	N/A	1.92 (1.3, 3)	Insufficient	Insufficient	3.96 (2.4, 5.9)	3.36 (2, 4.9)	N/A	3.24 (2.2, 4.9)	Sufficient	Sufficient
Morbidly Obese–pregnant	2	5.64 (3.5, 8.3)	4.92 (3, 7.3)	0.36	4.92 (3.1, 7.2)	Sufficient	Sufficient	7.32 (4.4, 10.6)	6.6 (4, 9.5)	1.8	6.48 (4.1, 9.4)	Sufficient	Sufficient
	4	4.08 (2.5, 6)	3.36 (2, 5.2)	N/A	3.36 (2.2, 5)	Sufficient	Sufficient	5.64 (3.5, 8.3)	4.92 (3, 7.3)	0.36	4.92 (3.1, 7.2)	Sufficient	Sufficient
	8	2.52 (1.56, 3.8)	**1.8 (1.1, 2.9)**	N/A	**1.8** (1.2, 2.8)	Insufficient	Insufficient	4.08 (2.5, 6)	3.36 (2, 5.2)	N/A	3.36 (2.2, 5)	Sufficient	Sufficient

Data in bracets represents 5th, 95th percentile

Table S3. Simulated percentages of obese– and morbidly obese–pregnant subjects achieving concentrations (total plasma, free plasma, adipose tissue homogenate, and adipose ISF) above minimum inhibitory concentration (2, 4 and 8 μg/mL or μg/g) at 1.5 hours and 2 hours post dose of 750 mg and 1500 mg

Table S4. Time above the minimum inhibitory concentration (2, 4 and 8 μg/mL) of different cefuroxime doses given virtually to obese–pregnant and morbidly obese–pregnant

**population**	**MIC_{90}**	**Simulated % of subjects achieved T>MIC_{90} (hours) post 750 mg cefuroxime dose at 1.5 hour (2 hours)**								**Simulated % of subjects achieved T>MIC_{90} (hours) post 1500 mg cefuroxime dose at 1.5 hour (2 hours)**							
		Plasma	Free plasma	Total adipose	ISF	Dose current dose achieve a targeted % of subjects attaining a target MIC, if cefuroxime were given before surgery at 30 or 60 min before surgery				Plasma	Free plasma	Total adipose	ISF	Dose current dose achieve a targeted % of subjects attaining a target MIC, if cefuroxime were given before surgery at 30 or 60 min before surgery			
						30 min or less		60 min						30 min or less		60 min	
						75 %	95 %	75 %	95 %					75 %	95 %	75 %	95 %
Obese–pregnant	2	100 (100)	100 (100)	0.4 (0)	100 (100)	Yes	Yes	Yes	Yes	100 (100)	100 (100)	84 (42)	100 (100)	Yes	Yes	Yes	Yes
	4	100 (99.6)	99.9 (97)	0 (0)	100 (97.2)	Yes	Yes	Yes	Yes	100 (100)	100 (100)	0.4 (0)	100 (100)	Yes	Yes	Yes	Yes
	8	98.6 (83.4)	84.6 (45.2)	0 (0)	85.7 (44.2)	Yes	No	No	No	100 (99.6)	99.9 (97)	0 (0)	100 (97.2)	Yes	Yes	Yes	Yes
Morbidly Obese–pregnant	2	100 (100)	100 (100)	0(0)	100 (100)	Yes	Yes	Yes	Yes	100 (100)	100 (100)	75.6 (34.2)	100 (100)	Yes	Yes	Yes	Yes
	4	100 (99.6)	99.9 (96.8)	0 (0)	100 (97.7)	Yes	Yes	Yes	Yes	100 (100)	100 (100)	0 (0)	100 (100)	Yes	Yes	Yes	Yes
	8	96.1 (79.5)	75.8 (37.5)	0 (0)	78.2 (36.2)	Yes	No	No	No	100 (99.6)	99.9 (96.8)	0 (0)	100 (97.7)	Yes	Yes	Yes	Yes

Dose (mg)	Time from administration to skin incision	MIC_{90} (μg/mL)	Obese–pregnant women		Morbidly obese–pregnant	
			T>MIC (hours)	*f*T>MIC (hours)	T>MIC (hours)	*f*T>MIC (hours)
750	15 min	2	5.03(3.15, 7.55)	4.43(2.75, 6.75)	5.39(3.25, 8.05)	4.67(2.75, 7.05)
		4	3.71(2.15, 5.63)	3.11(1.75, 4.67)	3.83(2.25, 5.75)	3.11(1.75, 4.95)
		8	2.27(1.45, 3.71)	1.67(0.95, 2.75)	2.27(1.31, 3.55)	1.55(0.85, 2.65)
	30 min	2	4.78(2.9, 7.3)	4.18(2.5, 6.5)	5.14(3, 7.8)	4.42(2.5, 6.8)
		4	3.46(1.9, 5.38)	2.86(1.5, 4.42)	3.58(2, 5.5)	2.86(1.5, 4.7)
		8	2.02(1.2, 3.46)	1.42(0.7, 2.5)	2.02(1.06, 3.3)	1.3(0.6, 2.4)
	1 h	2	4.28(2.4, 6.8)	3.68(2, 6)	4.64(2.5, 7.3)	3.92(2, 6.3)
		4	2.96(1.4, 4.88)	2.36(1, 3.92)	3.08(1.5, 5)	2.36(1, 4.2)
		8	1.52(0.7, 2.96)	0.92(0.2, 2)	1.52(0.56, 2.8)	0.8(0.1, 1.9)
1500	15 min	2	6.59(3.95, 9.35)	5.87(3.55, 8.65)	7.07(4.15, 10.35)	6.35(3.75, 9.25)
		4	5.03(3.15, 7.55)	4.43(2.75, 6.75)	5.39(3.25, 8.05)	4.67(2.75, 7.05)
		8	3.71(2.15, 5.65)	3.11(1.75, 4.65)	3.83(2.25, 5.75)	3.11(1.75, 4.95)
	30 min	2	6.34(3.7, 9.1)	5.62(3.3, 8.4)	6.82(3.9, 10.1)	6.1(3.5, 9)

	4	4.78(2.9, 7.3)	4.18(2.5, 6.5)	5.14(3, 7.8)	4.42(2.5, 6.8)
	8	3.46(1.9, 5.4)	2.86(1.5, 4.4)	3.58(2, 5.5)	2.86(1.5, 4.7)
1 h	2	5.84(3.2, 8.6)	5.12(2.8, 7.9)	6.32(3.4, 9.6)	5.6(3, 8.5)
	4	4.28(2.4, 6.8)	3.68(2, 6)	4.64(2.5, 7.3)	3.92(2, 6.3)
	8	2.96(1.4, 4.9)	2.36(1, 3.9)	3.08(1.5, 5)	2.36(1, 4.2)

MIC_{90} minimum inhibitory concentration required to inhibit the growth of 90% of organisms, *f*T>MIC time of free concentration above the minimum inhibitory concentration, T>MIC Time above the minimum inhibitory concentration. Numbers in brackets represent T>MIC and , *f*T>MIC of 5th, and 95th percentile.

Reference

1. Philipson, A.; Stiernstedt, G.; Ehrnebo, M. Comparison of the pharmacokinetics of cephradine and cefazolin in pregnant and non-pregnant women. *Clin. Pharmacokinet.* **1987**, *12*, 136-144, doi:10.2165/00003088-198712020-00004.
2. Rattie, E.S.; Ravin, L.J. Pharmacokinetic interpretation of blood levels and urinary excretion data for cefazolin and cephalothin after intravenous and intramuscular administration in humans. *Antimicrob. Agents Chemother.* **1975**, *7*, 606-613, doi:10.1128/aac.7.5.606.
3. Elkomy, M.H.; Sultan, P.; Drover, D.R.; Epshtein, E.; Galinkin, J.L.; Carvalho, B. Pharmacokinetics of prophylactic cefazolin in parturients undergoing cesarean delivery. *Antimicrob. Agents Chemother.* **2014**, *58*, 3504-3513, doi:10.1128/aac.02613-13.
4. Swank, M.L.; Wing, D.A.; Nicolau, D.P.; McNulty, J.A. Increased 3-gram cefazolin dosing for cesarean delivery prophylaxis in obese women. *Am. J. Obstet. Gynecol.* **2015**, *213*, doi:10.1016/j.ajog.2015.05.030.
5. Pevzner, L.; Swank, M.; Krepel, C.; Wing, D.A.; Chan, K.; Edmiston, C.E.J. Effects of maternal obesity on tissue concentrations of prophylactic cefazolin during cesarean delivery. *Obstet. Gynecol.* **2011**, *117*, 877-882, doi:10.1097/AOG.0b013e31820b95e4.
6. Chen, X.; Brathwaite, C.E.; Barkan, A.; Hall, K.; Chu, G.; Cherasard, P.; Wang, S.; Nicolau, D.P.; Islam, S.; Cunha, B.A. Optimal cefazolin prophylactic dosing for bariatric surgery: No need for higher doses or intraoperative redosing. *Obes. Surg.* **2017**, *27*, 626-629.
7. Chan, K.; Krepel, C.; Edmiston Jr, C.; Pevzner, L.; Swank, M.; Wing, D.A. Effects of maternal obesity on tissue concentrations of prophylactic cefazolin during cesarean delivery. *Am. J. Obstet. Gynecol.* **2011**, *204 (1 SUPPL.)*, S24, doi:http://dx.doi.org/10.1016/j.ajog.2010.10.052.
8. Stitely, M.; Sweet, M.; Slain, D.; Alons, L.; Holls, W.; Hochberg, C.; Briggs, F. Plasma and tissue cefazolin concentrations in obese patients undergoing cesarean delivery and receiving differing pre-operative doses of drug. *Surg. Infect. (Larchmt.)* **2013**, *14*, 455-459, doi:https://dx.doi.org/10.1089/sur.2012.040.
9. Young, O.M.; Shaik, I.H.; Twedt, R.; Binstock, A.; Althouse, A.D.; Venkataramanan, R.; Simhan, H.N.; Wiesenfeld, H.C.; Caritis, S.N. Pharmacokinetics of cefazolin prophylaxis in obese gravidae at time of cesarean delivery. *Am. J. Obstet. Gynecol.* **2015**, *213*, 541.e541-547, doi:https://dx.doi.org/10.1016/j.ajog.2015.06.034.
10. Groff, S.M.; Fallatah, W.; Yang, S.; Murphy, J.; Crutchfield, C.; Marzinke, M.; Kurtzberg, J.; Lee, C.K.K.; Burd, I.; Azadeh, F. Effect of maternal obesity on maternal-fetal transfer of preoperative cefazolin at cesarean section. *J Pediatr Pharmacol Ther* **2017**, *22*, 227-232, doi:10.5863/1551-6776-22.3.227.
11. Maggio, L.; Nicolau, D.P.; DaCosta, M.; Rouse, D.J.; Hughes, B.L. Cefazolin prophylaxis in obese women undergoing cesarean delivery: A randomized controlled trial. *Obstet. Gynecol.* **2015**, *125*, 1205-1210, doi:https://dx.doi.org/10.1097/AOG.0000000000000789.
12. Kram, J.J.F.; Greer, D.M.; Cabrera, O.; Burlage, R.; Forgie, M.M.; Siddiqui, D.S. Does current cefazolin dosing achieve adequate tissue and blood concentrations in obese women undergoing cesarean section? *Eur. J. Obstet. Gynecol. Reprod. Biol.* **2017**, *210*, 334-341, doi:https://dx.doi.org/10.1016/j.ejogrb.2017.01.022.
13. Lovering, A.M.; Perez, J.; Bowker, K.E.; Reeves, D.S.; MacGowan, A.P.; Bannister, G. A comparison of the penetration of cefuroxime and cephamandole into bone, fat and haematoma fluid in patients undergoing total hip replacement. *J. Antimicrob. Chemother.* **1997**, *40*, 99-104.
14. Kagedal, M.; Nilsson, D.; Huledal, G.; Reinholdsson, I.; Cheng, Y.-F.; Asenblad, N.; Pekar, D.; Borga, O. A study of organic acid transporter-mediated pharmacokinetic interaction between NXY-059 and cefuroxime. *J. Clin. Pharmacol.* **2007**, *47*, 1043-1049.
15. Garton, A.; Rennie, R.; Gilpin, J.; Marrelli, M.; Shafran, S. Comparison of dose doubling with probenecid for sustaining serum cefuroxime levels. *J. Antimicrob. Chemother.* **1997**, *40*, 903-906.
16. Hsu, V.; de, L.T.V.M.; Zhao, P.; Zhang, L.; Zheng, J.H.; Nordmark, A.; Berglund, E.G.; Giacomini, K.M.; Huang, S.M. Towards quantitation of the effects of renal impairment and probenecid inhibition on kidney uptake and efflux transporters, using physiologically based pharmacokinetic modelling and simulations. *Clin. Pharmacokinet.* **2014**, *53*, 283-293, doi:10.1007/s40262-013-0117-y.
17. Philipson, A.; Stiernstedt, G. Pharmacokinetics of cefuroxime in pregnancy. *Am. J. Obstet. Gynecol.* **1982**, *142*, 823-828, doi:https://doi.org/10.1016/S0002-9378(16)32526-1.
18. Bousfield, P.; Mullinger, B.M.; Elstein, M. Cefuroxime: Potential use in pregnant women at term. *Br. J. Obstet. Gynaecol.* **1981**, *88*, 146-149.

19. Lalic-Popovic, M.; Paunkovic, J.; Grujic, Z.; Golocorbin-Kon, S.; Milasinovic, L.; Al-Salami, H.; Mikov, M. Decreased placental and transcellular permeation of cefuroxime in pregnant women with diabetes. *J. Diabetes* **2016**, *8*, 238-245, doi:https://dx.doi.org/10.1111/1753-0407.12288.
20. Alrammaal, H.H.; Batchelor, H.K.; Morris, K.; Chong, H.P. Efficacy of perioperative cefuroxime as a prophylactic antibiotic in women requiring caesarean section: A systematic review. *European Journal of Obstetrics and Gynecology and Reproductive Biology* **2019**.
21. Barbour, A.; Schmidt, S.; Rout, W.R.; Ben-David, K.; Burkhardt, O.; Derendorf, H. Soft tissue penetration of cefuroxime determined by clinical microdialysis in morbidly obese patients undergoing abdominal surgery. *Int. J. Antimicrob. Agents* **2009**, *34*, 231-235, doi:10.1016/j.ijantimicag.2009.03.019.
22. Comer, J.; Tam, K. Lipophilicity profiles: theory and measurement. **2001**, 275-304.
23. Gaohua, L.; Abduljalil, K.; Jamei, M.; Johnson, T.N.; Rostami-Hodjegan, A. A pregnancy physiologically based pharmacokinetic (p-PBPK) model for disposition of drugs metabolized by CYP1A2, CYP2D6 and CYP3A4. *Br. J. Clin. Pharmacol.* **2012**, *74*, 873-885, doi:10.1111/j.1365-2125.2012.04363.x.
24. Abduljalil, K.; Furness, P.; Johnson, T.N.; Rostami-Hodjegan, A.; Soltani, H. Anatomical, physiological and metabolic changes with gestational age during normal pregnancy: a database for parameters required in physiologically based pharmacokinetic modelling. *Clin. Pharmacokinet.* **2012**, *51*, 365-396, doi:10.2165/11597440-000000000-00000.
25. Burt, H.J.; Neuhoff, S.; Almond, L.; Gaohua, L.; Harwood, M.D.; Jamei, M.; Rostami-Hodjegan, A.; Tucker, G.T.; Rowland-Yeo, K. Metformin and cimetidine: Physiologically based pharmacokinetic modelling to investigate transporter mediated drug–drug interactions. *Eur. J. Pharm. Sci.* **2016**, *88*, 70-82, doi:https://doi.org/10.1016/j.ejps.2016.03.020.
26. Neuhoff, S.; Gaohua, L.; Burt, H.; Jamei, M.; Li, L.; Tucker, G.T.; Rostami-Hodjegan, A. Accounting for transporters in renal clearance: Towards a mechanistic kidney model (Mech KiM). In *Transporters in Drug Development: Discovery, Optimization, Clinical Study and Regulation*, Sugiyama, Y., Steffansen, B., Eds.; Springer New York: New York, NY, 2013; pp. 155-177.
27. Abduljalil, K.; Pansari, A.; Jamei, M. Prediction of maternal pharmacokinetics using physiologically based pharmacokinetic models: assessing the impact of the longitudinal changes in the activity of CYP1A2, CYP2D6 and CYP3A4 enzymes during pregnancy. *J. Pharmacokinet. Pharmacodyn.* **2020**, *47*, 361-383, doi:10.1007/s10928-020-09711-2.
28. Ghobadi, C.; Johnson, T.N.; Aarabi, M.; Almond, L.M.; Allabi, A.C.; Rowland-Yeo, K.; Jamei, M.; Rostami-Hodjegan, A. Application of a systems approach to the bottom-up assessment of pharmacokinetics in obese patients. *Clin. Pharmacokinet.* **2011**, *50*, 809-822, doi:10.2165/11594420-000000000-00000.
29. Jamei, M.; Dickinson, G.L.; Rostami-Hodjegan, A. A framework for assessing inter-individual variability in pharmacokinetics using virtual human populations and integrating general knowledge of physical chemistry, biology, anatomy, physiology and genetics: A tale of 'bottom-up' vs 'top-down' recognition of covariates. *Drug Metab. Pharmacokinet.* **2009**, *24 1*, 53-75.
30. Spruill, W.J.; Wade, W.E.; Cobb, H.H., 3rd. Estimating glomerular filtration rate with a modification of diet in renal disease equation: implications for pharmacy. *Am. J. Health Syst. Pharm.* **2007**, *64*, 652-660, doi:10.2146/ajhp060239.
